# Stacking ensemble learning and SHAP analysis for predicting CO_2_ solubility and guiding ionic liquid design

**DOI:** 10.1098/rsos.260335

**Published:** 2026-10-07

**Authors:** Siqi Yu, Weiyan Tong, Pengzhi Bei, Hongwei Bai

**Affiliations:** ^1^ School of Chemical Process Automation, Shenyang University of Technology, Liaoyang, Liaoning, People's Republic of China; ^2^ School of Petrochemical Engineering, Shenyang University of Technology, Liaoyang, Liaoning, People's Republic of China

**Keywords:** stacking ensemble learning, ionic liquids, separation, CO_2_

## Abstract

Excessive CO_2_ emissions constitute a primary driver of global climate change, creating an urgent need for efficient CO_2_ capture technologies. Ionic liquids (ILs) are considered highly promising absorbents. However, their vast chemical space poses significant challenges for experimental screening. In this study, Morgan fingerprint descriptors were combined with a stacking ensemble learning algorithm, and SHapley Additive exPlanations (SHAP) analysis was employed to develop an accurate and interpretable framework for predicting CO_2_ solubility. The stacking ensemble model employs the light gradient boosting machine and the backpropagation neural network as base learners. The dung beetle optimizer and the grey wolf optimizer were used to determine optimal hyperparameter combinations for these learners, while linear regression was adopted as the meta-learner to produce the final CO_2_ solubility prediction. The results demonstrate that the stacking ensemble algorithm achieves the highest predictive accuracy, attaining a test-set coefficient of determination (*R*^2^) of 0.9888, a mean absolute error of 0.0149 and a mean squared error of 0.0006. SHAP analysis further reveals that the [Tf_2_N] (bis(trifluoromethylsulfonyl)amide) anion exerts the most significant influence, followed by the alkyl chain length of imidazolium cations. Accordingly, combining the [Tf_2_N] anion with imidazolium cations bearing longer alkyl chains represents a promising strategy for synthesizing ILs with high CO_2_ absorption efficiency.

## 1. Introduction

Global climate warming constitutes an urgent challenge that demands immediate international attention and concerted action [1]. As the primary greenhouse gas, CO_2_ plays a central role in driving climate change, and its excessive emissions pose serious threats to Earth's ecosystems by intensifying global warming and environmental degradation [2]. Developing effective methods to capture CO_2_, an acidic gas, and thereby mitigate its environmental impact has, therefore, become a worldwide priority. Based on their operating principles, CO_2_ capture technologies can be categorized into absorption, adsorption on solids [3], membrane separation and cryogenic separation [4]. Once captured, the CO_2_ can be sequestered by reaction with alkaline solid wastes to form stable carbonates [5]. Solid adsorption is frequently constrained by limited capacity and selectivity [6], while membrane separation faces challenges such as high material costs and sensitivity to gas composition [7]. Cryogenic separation, in turn, requires substantial energy input and complex infrastructure [8]. By contrast, absorption technology is widely adopted in industry owing to its maturity, adaptability and relatively high efficiency and selectivity for CO_2_ capture [9]. Nevertheless, conventional industrial absorbents continue to suffer from notable drawbacks. Inorganic alkaline solutions, including aqueous ammonia and carbonate systems, as well as amine-based solvents such as monoethanolamine and blended amines, all demand high regeneration energy and cause severe equipment corrosion [10]. Consequently, there is a pressing need to develop more environmentally benign, cost-effective and efficient absorbents to replace these traditional solvents. The past decade has witnessed growing interest in ionic liquids (ILs), a promising class of green solvents for CO_2_ capture. ILs are molten salts composed of organic cations and organic or inorganic anions, and they remain liquid at or near ambient temperature [11]. They possess a suite of favourable physico-chemical properties, including negligible vapour pressure, high thermal stability, low volatility and outstanding structural tunability. Research on ILs for CO_2_ absorption began in the early twenty-first century. A foundational study by Bates *et al.* [12] in 2002 first demonstrated the CO_2_ absorption capability of an IL, stimulating widespread investigation. Subsequent work has further advanced the field. For example, Yang *et al.* [13] employed a mixed-solvent system comprising 1-aminopropyl-1-methylimidazolium tetrafluoroborate ([Apmim][BF4]) and metal salts in a T-shaped microchannel reactor. This system achieved a volumetric mass transfer coefficient (K_L_a) significantly higher than that of conventional equipment. A defining advantage of ILs is their remarkable structural designability. By varying the cation and anion combinations, the number of theoretically accessible ILs is estimated to reach as high as 10^18^. While this vast combinatorial space offers immense potential for tailoring solvent properties, it also presents a formidable challenge, namely how to efficiently screen such enormous chemical diversity to identify optimal candidates for CO_2_ capture.

Therefore, developing models capable of rapidly and accurately predicting the CO_2_ solubility of ILs can not only greatly improve screening efficiency but also provide valuable guidance for the design and synthesis of novel ILs, thereby contributing to environmental protection and advances in gas treatment technologies. In this context, artificial intelligence, particularly machine learning, has emerged as a powerful tool. Machine learning excels at identifying complex patterns in high-dimensional data, accurately predicting physico-chemical properties and enabling inverse molecular design. It thus offers new solutions for the efficient screening and design of next-generation, high-performance absorbents and has been widely applied in energy materials [14], chemical engineering [15] and biomedicine [16]. Accurate and efficient prediction of CO_2_ solubility can be achieved by constructing quantitative structure–property relationship models, commonly referred to as QSPR models, that quantitatively link target properties with specific molecular descriptors. In 2022, Valeh-e-Sheyda *et al.* [17] developed feed-forward neural network (FFNN), radial basis function neural network and support vector machine (SVM) models based on 4397 CO_2_ solubility data points for 43 different imidazolium-based ILs. Using 26 descriptors as inputs, they found that the FFNN optimized by the Levenberg–Marquardt algorithm performed best, achieving an *R*^2^ of 0.9458. Venkatraman [18] collected 10 000 CO_2_ solubility data points for 185 ILs across different temperatures and pressures and employed semi-empirical geometric and charge-based molecular descriptors combined with a random forest (RF) model for prediction. Song *et al.* [19], drawing on 10 116 CO_2_ solubility data points for 124 ILs, proposed artificial neural network (ANN) and SVM models built upon a group contribution methodology. Tian *et al.* [20] assembled a dataset of 13 055 CO_2_ solubility data points for 164 ILs. They decomposed the ILs into 38 ionic fragments (IFs) and then integrated the IF contribution approach with SVM and ANN models. Kuroki *et al.* [21] applied density functional theory (DFT) to calculate geometric and electronic structure features of cations and anions for 402 114 ILs and built a Matérn 5/2 Gaussian process regression model to identify optimal cation–anion combinations. Their results indicated that [P_66614_][PFOS] (tetradecyltrihexylphosphonium perfluorooctanesulfonate) exhibits particularly high CO_2_ solubility. Zhang *et al.* [22] combined the NIST IL database with molecular dynamics (MD) simulations to construct a dataset of 216 CO_2_ Henry's law constants. They converted Simplified Molecular Input Line Entry System (SMILES) representations into 208 fixed molecular descriptors using RDKit and then built SVM, RF and multilayer perceptron (MLP) neural network models. Jian *et al.* [23] used 10 117 CO_2_ solubility data points for ILs under varying temperatures and pressures, generated 4096 Morgan fingerprint descriptors with RDKit and combined these descriptors with pressure and temperature as inputs to develop SVM, RF, eXtreme Gradient Boosting (XGBoost) and MLP models. Gao *et al.* [24] collected approximately 40 000 experimental data points, developed high-accuracy graph neural network regression and XGBoost classification models and, through high-throughput screening, successfully identified 12 ILs with the highest CO_2_ solubility. Ashraf *et al.* [25] investigated six advanced machine learning models using thermodynamic properties as input features and found that the deep gradient boosting machine (DeepGBM) achieved the highest accuracy. While conventional hyperparameter optimization methods, such as grid and random search, have been widely adopted, they face significant limitations in navigating high-dimensional, nonlinear and non-convex search spaces. Furthermore, prevailing CO_2_ solubility prediction models typically rely on single-algorithm frameworks. Although useful, such models often lack the robustness and accuracy required for high-dimensional, heterogeneous datasets.

To address the above issues, this paper proposes a practical prediction framework based on the stacking ensemble strategy for the specific application scenario of CO_2_ solubility prediction in ILs. The framework employs Morgan fingerprints as molecular descriptors, uses dung beetle optimizer-light gradient boosting machine (DBO-LGBM) and grey wolf optimizer-backpropagation neural network (GWO-BPNN) as base learners for initial predictions and selects linear regression (LR) as the meta-learner for result fusion. LGBM and BPNN can effectively capture linear and nonlinear feature relationships, respectively, while DBO and GWO achieve stable and efficient hyperparameter optimization. The stacking ensemble further integrates the complementary advantages of the base learners, thereby better capturing the complex correlations between cation/anion structures and dissolution behaviour. In addition, this study incorporates SHapley Additive exPlanations (SHAP) explainability analysis to elucidate how key chemical features influence carbon dioxide solubility, thereby providing theoretical guidance for the design and screening of novel high-performance ILs. By purposefully integrating existing methods and introducing rigorous validation strategies, such as structure-exclusive splitting and cross-system transfer tests, this work provides a high-accuracy, interpretable predictive tool for the fields of chemistry and engineering, reflecting an application-oriented integration innovation.

## 2. Material and methods

### 2.1. Experimental data

The dataset used in this study was collected and published by Lei *et al.* [26]. It consists of 10 116 CO_2_ solubility data points for 124 different ILs, including 28 anions and 56 cations, covering a temperature range of 243.2–453.15 K, a pressure range of 0.00798–499.9 bar and CO_2_ solubility values (mole fraction of CO_2_ in ILs) ranging from 0.0000648 to 0.9516. The structures of both cations and anions in the ILs are represented using SMILES notation. Table 1 lists a portion of the dataset.

**Table 1.** Sample data from the dataset.

IL	cation	anion	*T* (K)	*P* (bar)	solubility
[he][FOR]	OCC[NH_3_^+^]	C(=O)[O^−^]	323	95.3	0.2179
[MMIM][DMPO_4_]	C[N^+^]1 = CN(C = C1)C	COP(=O)(OC)[O^−^]	333.15	1.7464	0.01148
[HOC_2_MIM][PF_6_]	OCC[N^+^]1 = CN(C = C1)C	F[P^−^](F)(F)(F)(F)F	353.15	11.27	0.07
[BMIM][BF_4_]	C(CCC)[N^+^]1 = CN(C = C1)C	F[B^−^](F)(F)F	363.15	246	0.61
[BBIM][MeSO_3_]	C(CCC)[N^+^]1 = CN(C = C1)CCCC	CS(=O)(=O)[O^−^]	312.85	117	0.7322
[BMIM][Cl]	C(CCC)[N^+^]1 = CN(C = C1)C	[Cl^−^]	373.15	369.46	0.406
[C_3_OMIM][C(CN)_3_]	COCC[N^+^]1 = CN(C = C1)C	C(#N)[C^−^](C#N)C#N	323.15	16.93	0.1873
[OMIM][SCN]	C(CCCCCCC)[N^+^]1 = CN(C = C1)C	[S^−^]C#N	298.15	36.58	0.3481

Each data point in the dataset includes the SMILES of ILs’ cation and anion, temperature, pressure and CO_2_ solubility. The cations include imidazolium, pyrrolidinium, pyridinium, piperidinium, ammonium, phosphonium and sulfonium, while the anions comprise tetrafluoroborate ([BF_4_]), chloride ([Cl]), dicyanamide ([DCA]), nitrate ([NO_3_]), hexafluorophosphate ([PF_6_]), thiocyanate ([SCN]), tricyanomethanide ([C(CN)_3_]), bis(trifluoromethylsulfonyl)imide ([Tf_2_N]), methyl sulfate ([MeSO_4_]), among others. Figure 1 illustrates the structures of selected cations and anions of ILs in the dataset. For model training and evaluation, the dataset was randomly split into training, validation and test sets in a 7 : 1 : 2 ratio. The training set was used to learn model parameters, the validation set was used for hyperparameter tuning, and the test set was reserved for final model performance evaluation. The detailed partitioning information is summarized in table 2.

**Figure 1.**
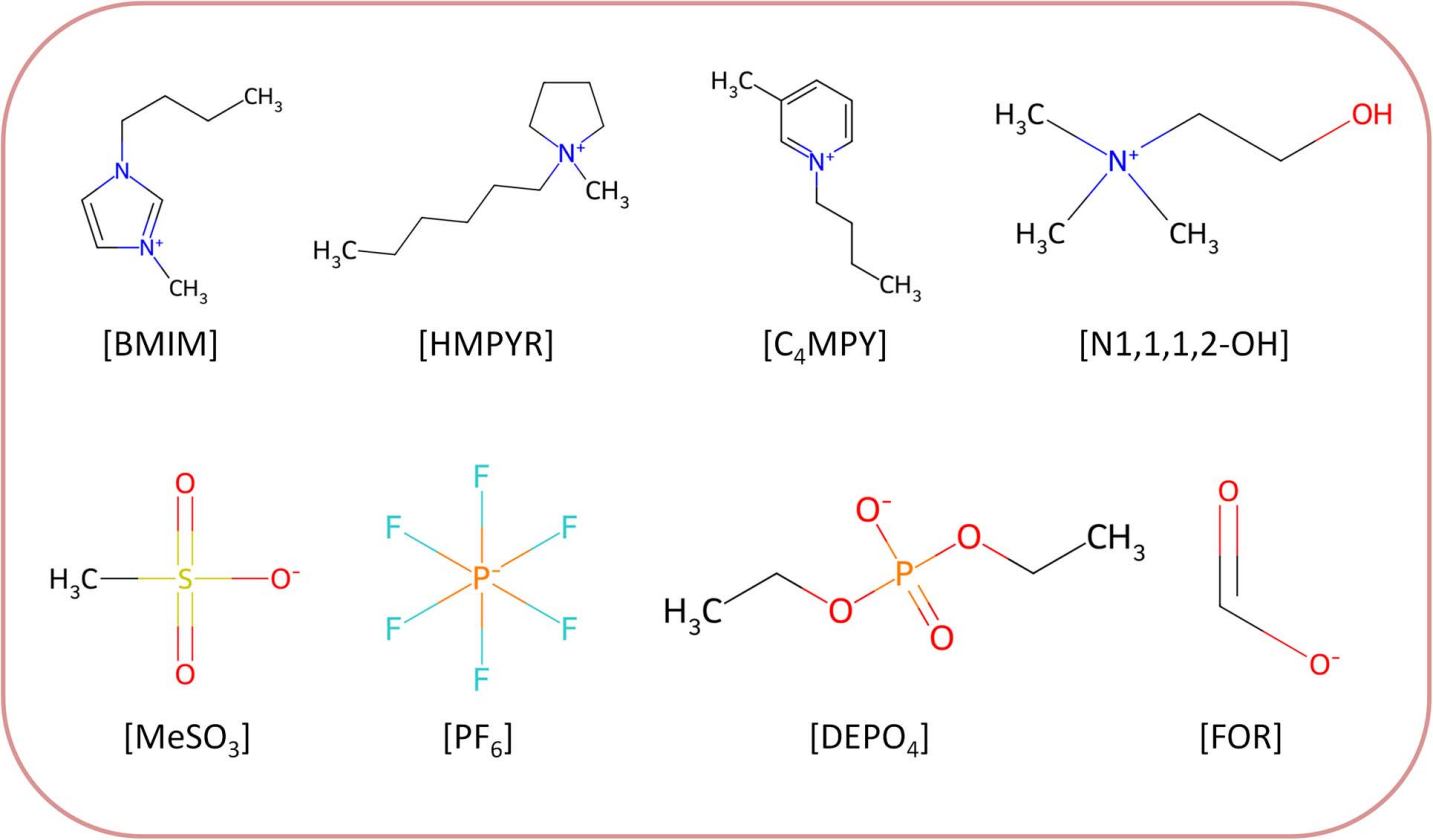
Cation and anion structures.

**Table 2.** Data partitioning information.

dataset	data point count	proportion (%)
training set	7080	70
validation set	1012	10
test set	2024	20
total	10 116	100

To more clearly illustrate the dataset's composition, both cations and anions were classified and counted by chemical family. Tables 3 and 4 list the specific ion species contained in each category, as well as their sample number distributions across the training, validation and test sets, thereby reflecting the coverage and diversity of the dataset across different structural types.

**Table 3.** Cation classification and data distribution.

types	cation	training	validation	test
imidazolium	[MMIM], [EMIM], [PMIM], [BMIM], [C_5_MIM], [HMIM], [C_7_MIM], [OMIM], [NMIM], [DMIM], [C_12_MIM], [C_6_F_9_MIM], [C_8_F_13_MIM], [HOC_2_MIM], [HOC_3_MIM], [C_3_OMIM], [C_5_O_2_MIM], [aMIM], [oleyl-MIM], [BBIM], [(ETO)_2_IM], [BMMIM], [HMMIM]	5564	816	1587
pyrrolidinium	[C_3_MPYR], [BMPYR], [C_5_MPYR], [HMPYR], [C_7_MPYR], [OMPYR], [C_9_MPYR], [COCMPYR]	674	80	203
pyridinium	[C_4_PY], [HOEPY], [PMPY], [C_4_MPY], [HMPY]	110	17	35
piperidinium/piperazine	[PMPip], [he], [hea], [thma], [deme], [MDEA], [m-2-HEA]	185	27	54
quaternary ammonium/phosphonium/sulfonium	[N_1,1,1,2_–OH], [N_1,1,3,2_–OH], [N_1,8,8,8_], [N_2,1,1,3_], [N_4,1,1,1_], [N_1,4,4,4_], [N_4,4,4,4_], [N_6,1,1,1_], [P_1,4,4,4_], [P_2,4,4,4_], [P_4,4,4,4_], [P_6,6,6,14_], [S_222_]	547	72	145

**Table 4.** Anion classification and data distribution.

types	anion	training	validation	test
halide/inorganic	[BF_4_], [Cl], [DCA], [NO_3_], [PF_6_], [SCN], [TCB], [C(CN)_3_], [HSO_4_]	2641	400	765
organic sulfonate/fluorosulfonate	[FSA], [Tf_2_N], [BETA], [FOR], [TFA], [C_3_F_7_CO_2_], [MeSO_4_], [EtSO_4_], [MDEGSO_4_], [MeSO_3_], [TfO], [NfO], [TDfO], [TOS], [C_12_PhSO_3_]	4362	604	1236
phosphate	[DMPO_4_], [DEPO_4_], [DBPO_4_]	62	7	18
other special anions	[methide]	15	1	5

### 2.2. Morgan fingerprint

To accurately represent the structural information of ILs and precisely predict CO_2_ solubility, Morgan fingerprints were selected as molecular descriptors [27]. Morgan fingerprints describe molecular features by converting molecular structures into fixed-length binary bit vectors. Their generation process is similar to that of topological fingerprints and can be summarized in four steps: (i) initial assignment of atom identifiers: an initial identifier is assigned to each non-hydrogen atom to capture local atomic information, including basic chemical properties such as atom type and atomic charge; (ii) iterative identifier generation: during the iterative process, the initial atom identifiers are combined with those of neighbouring atoms to capture progressively larger circular neighbourhoods, which are then transformed into new integer identifiers. These identifiers are collected as features describing local molecular substructures. Figure 2a illustrates the generation of Morgan fingerprint identifiers, where the blue circle represents the central atom and the asterisk (*) denotes the larger molecular structure connected to it; (iii) removal of duplicate identifiers: if two atomic neighbourhoods share the same set of chemical bonds or identical hash identifiers, they are considered equivalent, and redundant identifiers are removed to eliminate duplication; and (iv) generation of fingerprint bit strings: the identifiers are hashed and converted into a fixed-length bit string. The overall generation process of Morgan fingerprints is shown in figure 2b.

**Figure 2.**
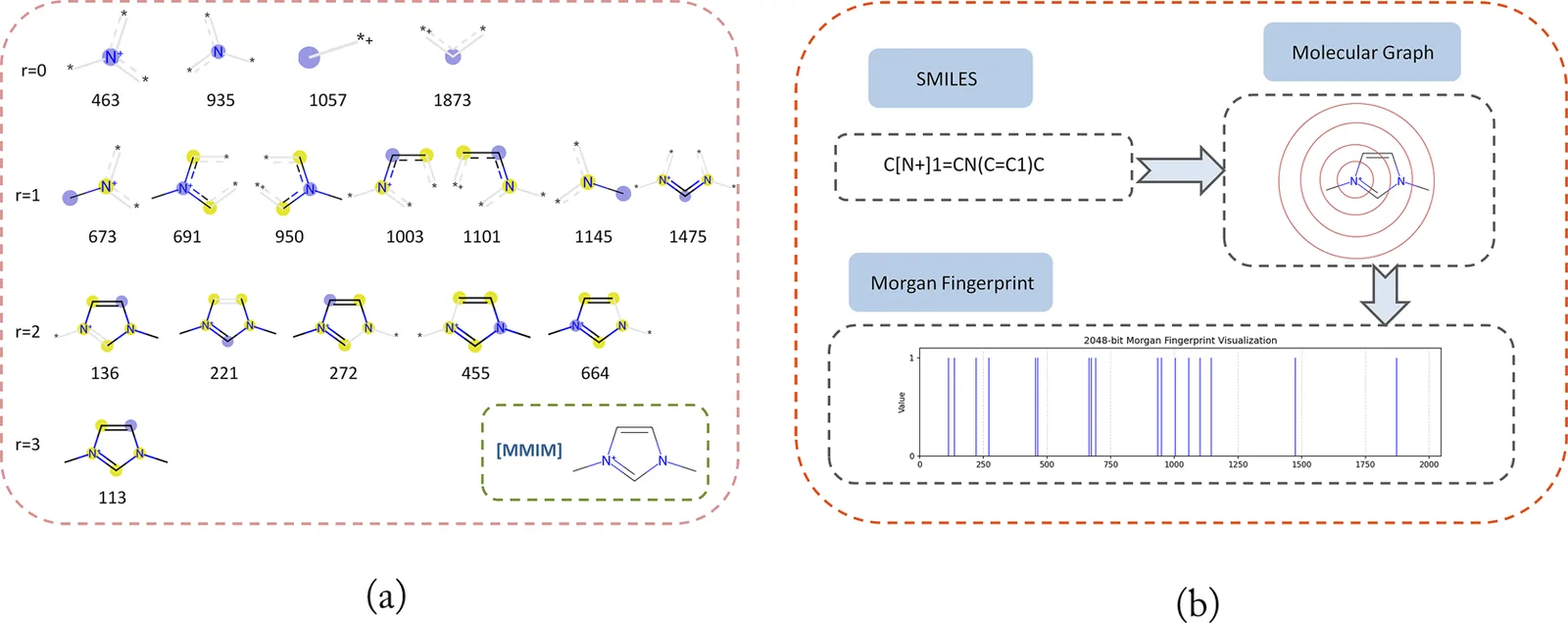
Morgan fingerprint generation process diagram: (a) example of Morgan fingerprint identifier [MMIM] and (b) Morgan fingerprint generation steps.

In this work, Morgan fingerprints with a radius of 3 and a bit length of 2048 were generated separately for the cations and anions. The fingerprints of the cation and anion for each IL were then concatenated into a 4096-dimensional vector. Owing to the extremely high dimensionality of this vector, variance analysis and Pearson correlation coefficients were employed to screen the fingerprint features. Ultimately, 211 Morgan fingerprint features were retained and, together with temperature and pressure, served as the input features for the predictive models.

### 2.3. Dung beetle optimizer-light gradient boosting machine

LGBM [28] is an efficient, improved algorithm for gradient boosting decision trees. It employs gradient-based one-sided sampling and exclusive feature bundling techniques, combined with an improved histogram algorithm and a leaf-wise growth strategy. In terms of computational efficiency, LGBM significantly reduces training time and memory consumption in high-dimensional feature spaces while maintaining high predictive accuracy, making it particularly suitable for large-scale, multivariate nonlinear regression tasks. For predicting CO_2_ solubility in ILs, this method effectively handles the high-dimensional, sparse structural descriptors derived from Morgan fingerprints and captures the complex nonlinear structure–property relationships, thereby enabling high-precision modelling of CO_2_ dissolution behaviour.

LGBM is an iterative learning method that gradually improves performance through successive iterations. In each iteration, a weak learner is obtained by minimizing the iterative loss function *L*[*y*, *F_t_*(*x*)], expressed as

(2.1) $$L\left[y,F_{t}\left(x\right)\right]=L\left[y,F_{t-1}\left(x\right)+h_{t}\left(x\right)\right],$$

where *F*_*t* − 1_(*x*) and *L*[*y*, *F*_*t* − 1_(*x*)] denote the strong learner and the loss function obtained in the previous iteration, and *h_t_*(*x*) represents the weak learner generated in the *t*th iteration.

Use the following formula to approximate the loss $r_{i}^{t}$ in this iteration using the negative gradient:

(2.2) $$r_{i}^{t}=\frac{\partial L\left[y_{i},F_{t-1}\left(x_{i}\right)\right]}{\partial F_{t-1}\left(x_{i}\right)}.$$

The square difference is used for the approximation fitting:

(2.3) $$h_{t}\left(x\right)=\underset{h\in H}{\arg\min}\sum_{i=1}^{n}{\left[r_{i}^{t}-h_{t}\left(x_{i}\right)\right]}^{2},$$

where *H* represents the function space of the learner, *n* is the total number of samples, and *h_t_*(*x_i_*) is the prediction value of the weak learner for the *t*th sample *x_i_*.

The final strong learner *F_t_*(*x*) obtained is

(2.4) $$F_{t}\left(x\right)=F_{t-1}\left(x\right)+h_{t}\left(x\right).$$

However, the LGBM model involves multiple hyperparameters, and complex nonlinear interactions among them significantly affect the model's ability to fit and generalize. Therefore, proper hyperparameter optimization is crucial for improving prediction accuracy. The DBO [29], as a novel swarm intelligence optimization algorithm, can achieve a good balance between global exploration and local exploitation through a multi-strategy search mechanism, thereby improving the efficiency and stability of solving complex continuous optimization problems. Based on these characteristics, this study employs DBO to automatically tune key hyperparameters of LGBM, thereby avoiding the limitations of manual tuning and further enhancing the model's overall performance and generalization in CO_2_ solubility prediction tasks.

DBO is a novel swarm intelligence optimization algorithm inspired by the biological behaviours of dung beetles, including ball-rolling, dancing, foraging, stealing and reproduction. By simulating these behaviours, the algorithm performs global exploration and local exploitation, ultimately obtaining the optimal solution, that is the best combination of hyperparameters.

*Dung beetle rolling:* dung beetles use celestial navigation, and their rolling path is influenced by the environment. During the rolling process, the change in the dung beetle's position is given by the following:

(2.5)xi(t+1)=xi(t)+α×k×xi(t−1)+b×Δx(2.6)Δx=|xi(t)−Xω|,

where *t* is the iteration number, *x_i_*(*t*) is the position of the *i*th dung beetle at the *t*th iteration, *k* ∈ (0, 0.2], *b* ∈ (0, 1);α = ±1,*X*^ω^ is the global worst position, and Δ*x* is used to simulate changes in light intensity.

*Dung beetle dancing:* when a dung beetle encounters an obstacle and cannot move forward, it obtains a new trajectory through dancing behaviour. The tangent function is used to determine a new path within the range of [0, π], allowing the rolling behaviour to continue:

(2.7) $$x_{i}\left(t+1\right)=x_{i}\left(t\right)+\tan\left(\theta \right)\left|x_{i}\left(t\right)-x_{i}\left(t-1\right)\right|.$$

*Dung beetle reproduction:* to ensure a safe oviposition site for dung beetles, the spawning area for female dung beetles is simulated based on a boundary selection strategy as follows:

(2.8)Lb∗=max(X∗×(1−R),Lb)(2.9)Ub∗=min(X∗×(1+R),Ub).

The position of the breeding dung beetle changes with the variation of the spawning area, and the position update is given by the following:

(2.10) $$B_{i}\left(t+1\right)=X^{*}+b_{1}\times \left(B_{i}\left(t\right)-Lb^{*}\right)+b_{2}\times \left(B_{i}\left(t\right)-Ub^{*}\right).$$

*Dung beetle foraging:* as small dung beetles grow, they emerge from the ground to search for food. The boundary range of the optimal foraging area is dynamically adjusted based on the global optimal position, and the position is updated as follows:

(2.11)Lbb=max(Xb×(1−R),Lb),(2.12)Ubb=min(Xb×(1+R),Ub)(2.13)xi(t+1)=xi(t)+C1×(xi(t)−Lbb)+C2×(xi(t)−Ubb).

*Dung beetle stealing:* some dung beetles steal dung balls from others. The position update for the thief dung beetle is given by the following:

(2.14) $$x_{i}\left(t+1\right)=X^{b}+S\times g\times \left(\left|x_{i}\left(t\right)-X^{*}\right|+\left|x_{i}\left(t\right)-X^{b}\right|\right).$$

Introducing stealing behaviour can further enhance the algorithm's search capability.

The procedure is as follows: (i) initialize the position of each dung beetle (i.e. the initial values of LGBM hyperparameters) and its fitness value. The population size is set to 20, and the maximum number of iterations is 30; (ii) for each dung beetle individual, train the LGBM model using the hyperparameter combination corresponding to its current position and calculate the model fitness; (iii) update the positions of dung beetles according to foraging, rolling and stealing behaviours. If the fitness at the new position is better than that at the original position, update the current position and its fitness; otherwise, retain the original position. These steps are repeated until the maximum number of iterations is reached; and (iv) output the optimal hyperparameters and pass them to the LightGBM model for training. The DBO process diagram is shown in figure 3.

**Figure 3.**
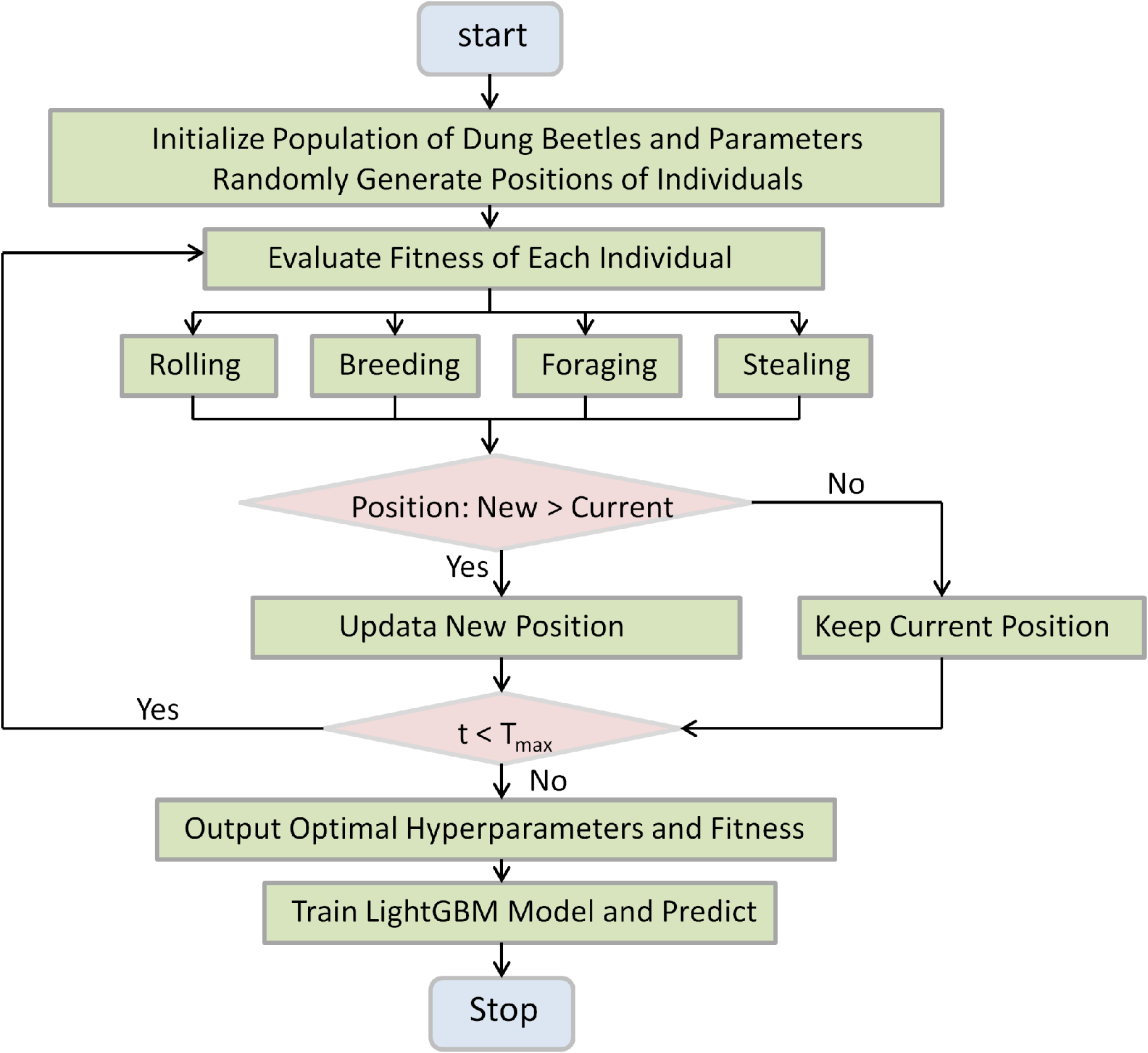
Dung beetle optimizer flow chart.

Table 5 lists the optimal hyperparameter combinations for the LGBM model obtained using the DBO algorithm.

**Table 5.** LGBM model hyperparameters.

hyperparameters	scope	values
n_estimators	(10, 300)	300
num_leaves	(3, 60)	45
max_depth	(2, 20)	20
learning_rate	(0.01, 0.3)	0.1695
random_state	–	42

### 2.4. Grey wolf optimizer-backpropagation neural network

BPNN [30] is a typical multilayer FFNN model composed of an input layer, hidden layers and an output layer. It is trained through an iterative mechanism of forward propagation and error backpropagation to continuously optimize network weights and minimize prediction error. In nonlinear function approximation, BPNN exhibits strong self-learning and nonlinear mapping capabilities, enabling it to effectively capture higher-order nonlinear relationships between complex input features and target variables. It also shows good fitting performance for multidimensional continuous variables. For the CO_2_ solubility prediction in ILs, BPNN can fully exploit the complex coupling relationships between Morgan molecular fingerprints and operating conditions (such as temperature and pressure), thereby enabling high-accuracy modelling of CO_2_ dissolution behaviour. The structure is shown in figure 4.

**Figure 4.**
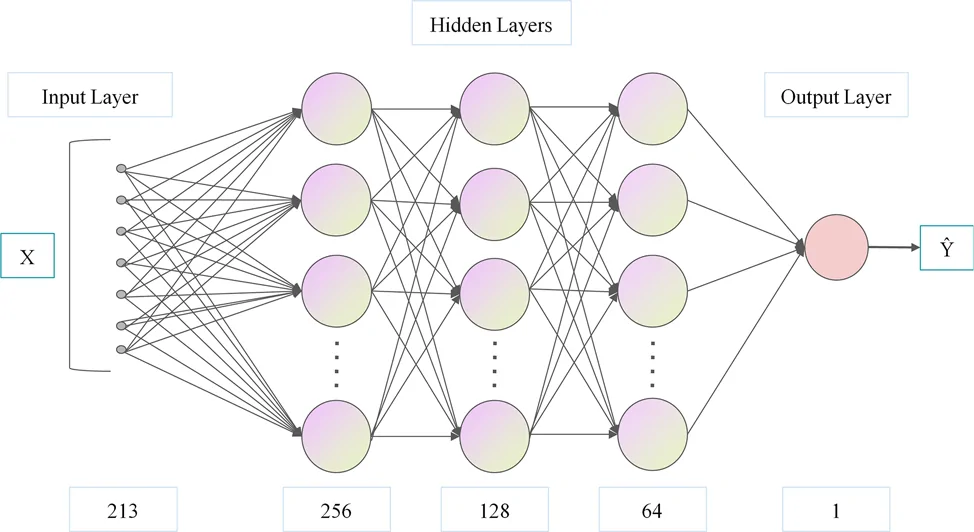
BPNN model structure diagram.

During forward information propagation, the input data are transmitted from the input layer to the hidden layers and then to the output layer. The output of the hidden layer can be expressed as

(2.15) $$H_{j}=f\left(\sum_{i=1}^{n}W_{ij}X_{i}-a_{j}\right),$$

where *f* denotes the activation function of the hidden-layer neurons; the ReLU function is selected as the activation function.

Subsequently, the output of the neural network is calculated using the weights and biases of the output layer:

(2.16) $$O_{k}=f\left(\sum_{j=1}^{l}H_{j}W_{jk}-b_{k}\right).$$

After obtaining the output, the error *e_k_* between the expected output and the actual output of the neural network is calculated as

(2.17) $$e_{k}=Y_{k}-O_{k}.$$

During backward information propagation, the error between the predicted and actual results is propagated backwards, and the weights and biases are continuously updated to minimize the error:

(2.18)W′ij=Wij+φHj(1−Hj)Xi∑k=1mWjkek,(2.19)a′j=aj+φHj(1−Hj)∑k=1mWjkek,(2.20)W′jk=Wjk+φHjek(2.21)b′k=bk+ek.

To determine the optimal hyperparameters of the BPNN model and enhance its predictive accuracy, the GWO [31] is employed to optimize the hyperparameters of BPNN. The *R*^2^ is used as the fitness function, and the objective function value is evaluated on the validation set to obtain the optimal parameter combination for predicting CO_2_ solubility. Compared with traditional random search methods, GWO offers advantages such as a simple structure, few parameters, fast convergence and strong global search capability, effectively reducing the dependence of the optimization process on initial conditions and improving the solution stability for complex non-convex optimization problems. For the hyperparameter optimization of BPNN, the search space is characterized by high nonlinearity, multimodality and strong coupling, making traditional methods prone to local optima or slow convergence. By contrast, GWO, through its multi-leader-guided collaborative search mechanism, enhances local fine-tuning ability while ensuring global exploration, thereby more effectively obtaining the parameter combination for BPNN and significantly improving the model's fitting accuracy and generalization capability for the task of CO_2_ solubility prediction.

The GWO, proposed by Mirjalili *et al.* in 2014 [31], is a swarm intelligence optimization algorithm inspired by the hunting behaviour of grey wolves, simulating their cooperative hunting strategy to solve optimization problems. The wolf pack is divided into four hierarchical groups according to social dominance: *α*, *β*, *δ* and *ω*. Among them, *α* denotes the best solution, *β* and *δ* denote the second- and third-best solutions, respectively, and *ω* denotes the set of candidate solutions. The implementation process of the GWO algorithm is as follows:

*encircling prey*:

(2.22)D=|C⋅Xp(t)−X(t)|,(2.23)X(t+1)=Xp(t)−A⋅D,(2.24)A=2a(r1−1)(2.25)C=2r2,

where *t* is the current iteration number, *X_p_*(*t*) and *X*(*t*) are the position vectors of the prey and the grey wolf, respectively, *A* and *C* are coefficient vectors, *r*_1_ and *r*_2_ are random number vectors within the range [0, 1], and *a* is the convergence factor.

*Hunting:* in each iteration, the positions of the *α*, *β* and *δ* wolves are considered the optimal positions, and the positions of other individual grey wolves are updated accordingly. The mathematical expressions for the update process are as follows:

(2.26)Dα=|C1Xα−X|,(2.27)Dβ=|C2Xβ−X|,(2.28)Dδ=|C3Xδ−X|,(2.29)X1=Xα−A1Dα,(2.30)X2=Xβ−A2Dβ,(2.31)X3=Xδ−A3Dδ(2.32)X(t+1)=X1+X2+X33,

where *A*_1_,*A*_2_,*A*_3_ and *C*_1_,*C*_2_,*C*_3_ represent the random disturbance value.

*Attacking prey:* when the wolves approach the prey within a certain distance, they initiate an attack. When |*A*| > 1 is satisfied, the grey wolves disperse to search for prey (global search); when |*A*| < 1 is satisfied, they move closer to the prey (local search). Finally, the fitness value of the alpha wolf is obtained as the optimal solution.

The procedure for optimizing the BPNN using GWO is as follows: (i) initialize the positions of grey wolves, with a population size of 10 and a maximum of 30 iterations; (ii) calculate the fitness of each individual and identify the positions of the *α*, *β* and *δ* wolves; (iii) update the positions of all wolves in the pack and recalculate their fitness values; (iv) check whether the maximum number of iterations has been reached. If so, the position of the *α* wolf is regarded as the optimal solution, that is the optimal hyperparameter combination; otherwise, return to step (ii); and (v) output the optimal hyperparameter combination and train the BPNN model. The GWO process diagram is shown in figure 5.

**Figure 5.**
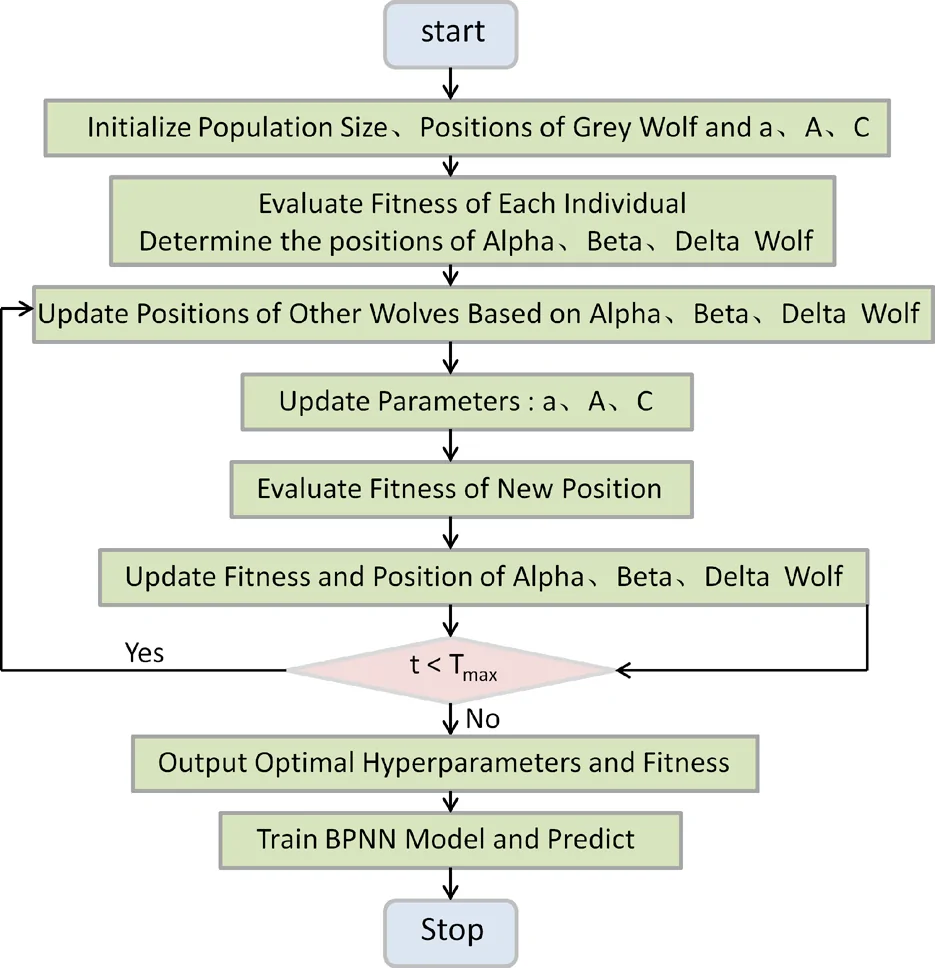
Grey wolf optimizer flow chart.

The optimal hyperparameter for the BPNN model is shown in table 6.

**Table 6.** BPNN model hyperparameters.

hyperparameters	scope	values
dropout_rate	(0.1, 0.5)	0.1
batch_size	[32 , 64, 128]	64
weight_decay	(1e−6, 1e−2)	1e−6
learning_rate	(1e−5, 1e−2)	0.002895

In this study, the neural network consists of five layers, including three hidden layers with 256, 128 and 64 neurons, respectively, and ReLU activation functions. The input layer comprises 213 features, including Morgan fingerprints, temperature and pressure, while the output layer employs a linear activation function to output the predicted CO_2_ solubility. The dropout rate of the hidden layers is set to 0.1. During training, 10% of the input units are randomly set to zero at each update, reducing the model's sensitivity to specific neurons with certain weights and thereby preventing overfitting. Mini-batch gradient descent is adopted with a batch size of 64, and the initial learning rate is set to 0.002895. Cosine annealing learning rate scheduling strategy is applied during training to dynamically adjust the learning rate and improve convergence efficiency. The Adam optimizer is used to update the network weights and biases to minimize the loss function.

### 2.5. Stacking ensemble learning

To address the limitations of single models in predicting CO_2_ solubility accuracy and robustness, the stacking ensemble algorithm provides an effective solution [32]. As a typical two-layer heterogeneous ensemble method, its core idea lies in exploiting the diversity and independence of base learners. On the one hand, each base learner must possess strong predictive capability to make meaningful contributions to the ensemble; on the other hand, diversity among models is emphasized to reduce error correlation. Through the stacking approach, different representations and processing mechanisms of individual models can be fully used to capture multi-perspective features of the data, thereby improving predictive accuracy and generalization performance while effectively avoiding overfitting.

In this work, the first layer of the stacking framework consists of two complementary base learners, DBO-LGBM and GWO-BPNN, whose predictions are combined into meta-features. The second layer uses LR as the meta-learner to learn from these features and generate the final prediction. The overall workflow is illustrated in figure 6. The specific steps of the stacking ensemble are as follows: (i) perform 10-fold cross-validation on the training set. The training data are divided into 10 equal subsets; in each fold, nine subsets are used for training and the remaining one for validation. Taking one fold as an example, the base learners are trained on the training data to obtain their predictions on the validation set *Y_t_* and on the test-set *U_t_*, where *Y_t_* and *U_t_* denote the *t*th base learner; (ii) concatenate the prediction values on the validation set column-wise to form the training samples of the meta-learner *Y_t_*′, and average the predictions on the test set to form the test samples of the meta-learner *U_t_*′; (iii) after repeating steps (i) and (ii) for all base learners, horizontally concatenate the resulting meta-learner training and test samples to obtain the final training and test sets for the meta-learner; and (iv) train and test the meta-learner using the dataset obtained in step (iii) to produce the final prediction results.

**Figure 6.**
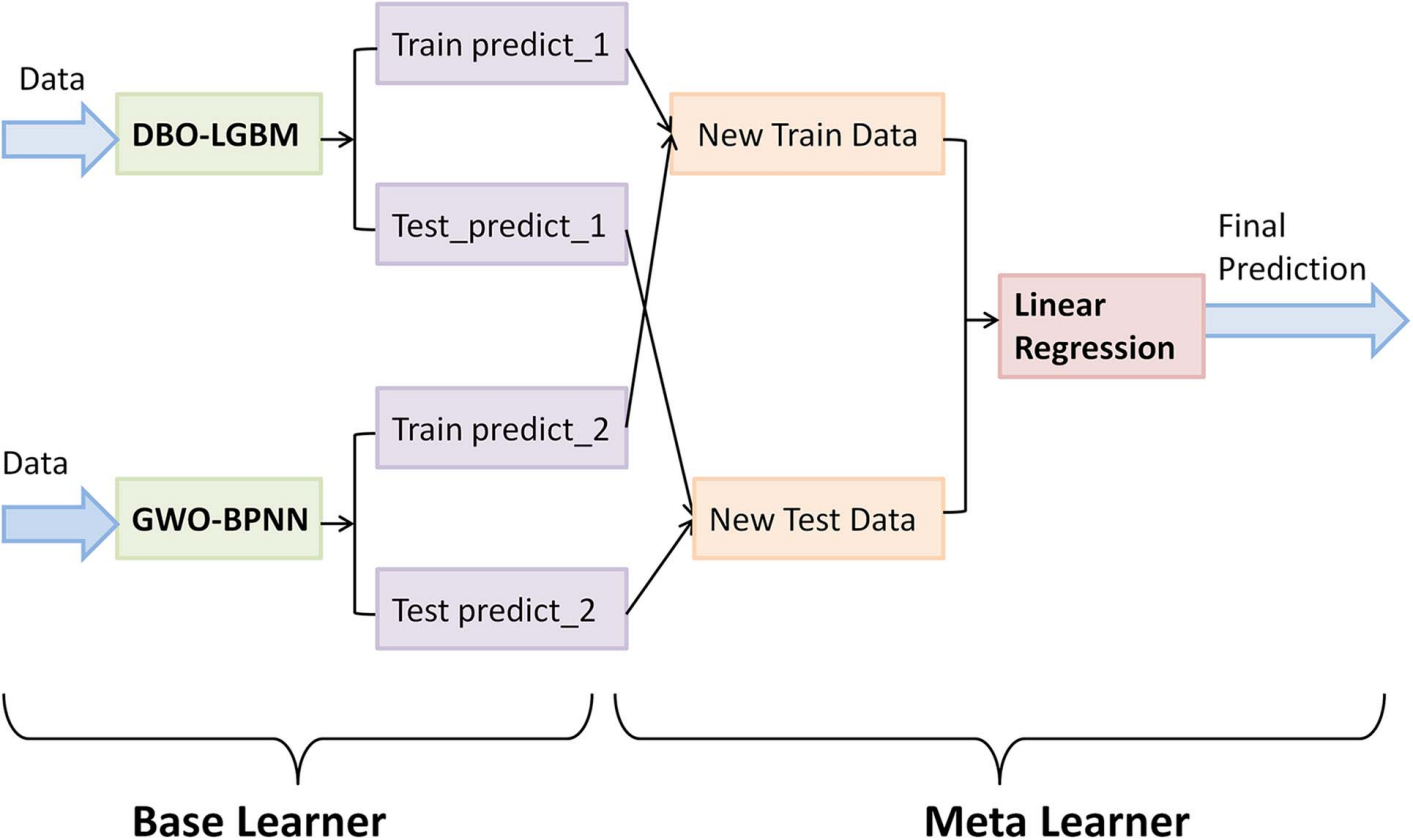
Stacking ensemble model.

### 2.6. Model evaluation metrics

To evaluate the performance of different predictive models, we use the mean absolute error (MAE), mean squared error (MSE) and coefficient of determination (*R*^2^) as evaluation metrics to assess prediction accuracy on both the training and test sets. These metrics collectively form the basis for evaluating and comparing the predictive accuracy and fitting quality of the models. Their calculation formulas are given as follows:

(2.33)MAE=1n∑i=1n|yi−y^l|,(2.34)MSE=1n∑i=1n(yi−y^l)2(2.35)R2=1−∑i=1n(yi−y^l)2∑i=1n(yi−y¯)2,

where *n* is the number of observations in the dataset, *y_i_* denotes the actual value, ${\hat{y}}_{l}$ represents the predicted value generated by the model, $\bar{y}$ is the mean of the actual values, and $\left|y_{i}-{\hat{y}}_{l}\right|$ is the absolute error of each prediction.

### 2.7. Modelling process

This study aims to develop a prediction and analysis framework based on stacked ensemble learning to predict CO_2_ solubility in ILs and to identify and analyse the contributions of key substructures to that solubility. The specific steps are as follows:

(i) data collection: the dataset is obtained from the previous work of Lei *et al.* and includes the SMILES of IL cations and anions, experimental conditions (temperature and pressure) and CO_2_ solubility (mole fraction of CO_2_ in ILs);
(ii) feature extraction: the original chemical molecular data are transformed into feature vectors suitable for model training. The RDKit is used to parse SMILES strings and convert them into structured molecular objects. Morgan fingerprints with a radius of 3 and a length of 2048 bits are generated to encode local molecular structural features based on atomic environment similarity;
(iii) construction of the stacking ensemble model: based on the stacking ensemble learning strategy, DBO-LGBM and GWO-BPNN are selected as base learners in the first layer, while LR is used as the meta-learner in the second layer. The stacking ensemble model is constructed to predict the solubility of CO_2_ in ILs; and
(iv) SHAP interpretability analysis: to uncover the underlying physico-chemical mechanisms embedded in the model, SHAP interpretability analysis is further conducted to reveal the contributions of structural features of ILs to CO_2_ solubility. This analysis helps identify the causal relationships between IL structures and CO_2_ solubility, thereby providing theoretical support for the design of highly efficient ILs.

The overall workflow is illustrated in figure 7.

**Figure 7.**
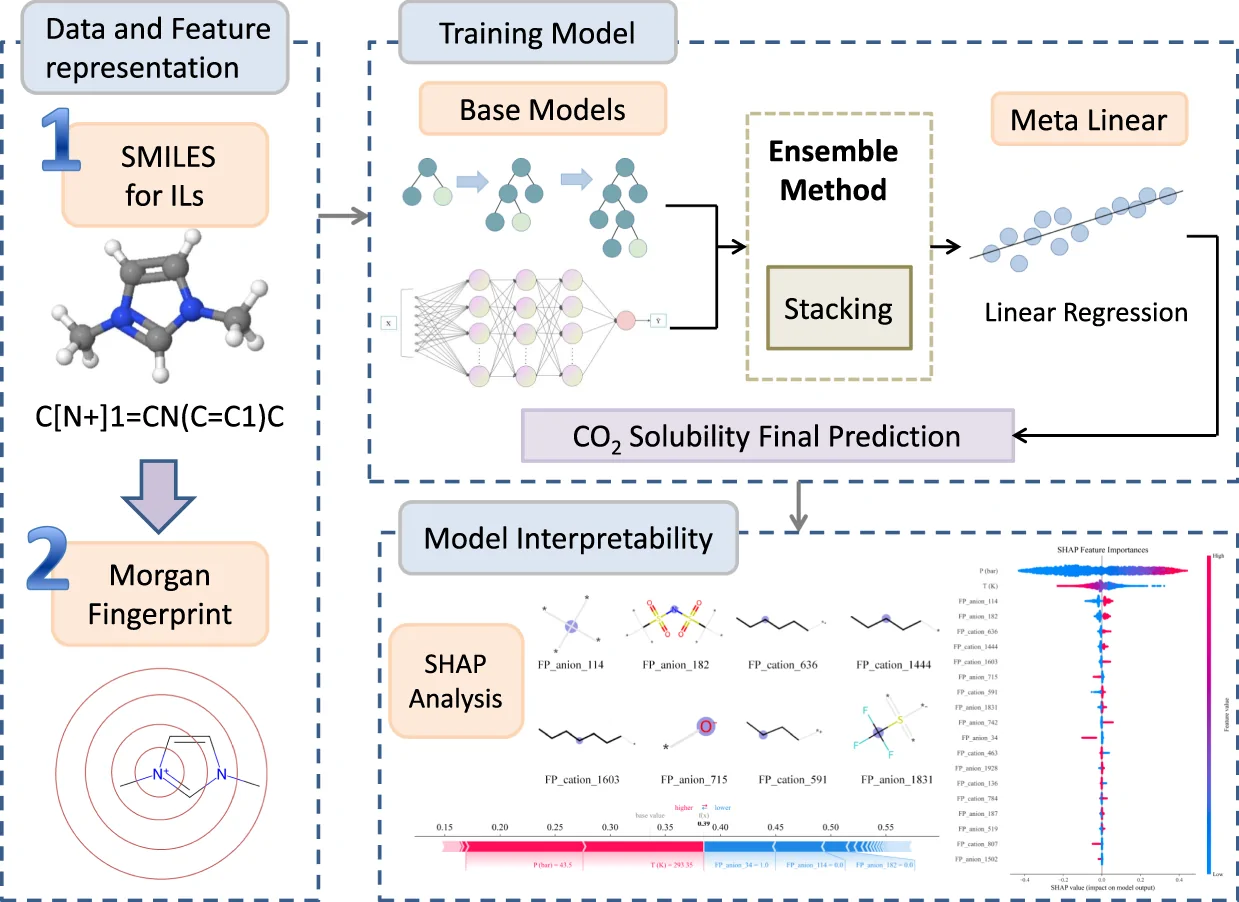
Technical roadmap for this article.

## 3. Results and discussion

### 3.1. Ablation experiment

To assess the performance of the stacking ensemble model, we compared its predictions with those of its base learners (DBO-LGBM and GWO-BPNN) and with the stand-alone LGBM and BPNN models. The predictive accuracy of each model is quantified using MAE, MSE and *R*^2^ on both training and test sets, with detailed results provided in table 7.

**Table 7.** Ablation experiment results of model performance on training and test sets.

	MAE		MSE		*R* ^2^	
method	training	test	training	test	training	test
LGBM	0.0164	0.0203	0.0006	0.0009	0.9892	0.9835
BPNN	0.0233	0.0262	0.0010	0.0013	0.9819	0.9770
DBO-LGBM	0.0091	0.0166	0.0002	0.0007	0.9963	0.9872
GWO-BPNN	0.0122	0.0154	0.0004	0.0007	0.9921	0.9878
stacking	0.0112	0.0149	0.0003	0.0006	0.9932	0.9888

The results demonstrate that incorporating the DBO and GWO optimization algorithms further improves the performance of the base learners, yielding test-set *R*^2^ values of 0.9872 for DBO-LGBM and 0.9878 for GWO-BPNN. The stacking ensemble model exhibits the best overall performance, achieving an *R*^2^ of 0.9888, an MAE of 0.0149 and an MSE of 0.0006 on the test set. All evaluation metrics surpass those of the other models, confirming that the stacking model provides the most accurate prediction of CO_2_ solubility in ILs. This superiority arises from the fundamental differences between LGBM, a tree-based model, and BPNN, a neural network-based model, in their functional forms and data-fitting mechanisms. These two learners approximate the true distribution of CO_2_ solubility from distinct perspectives, leading to weakly correlated prediction errors. By effectively integrating the complementary information captured by both base learners, the stacking model enhances diversity and robustness, thereby improving overall generalization performance.

To further illustrate the model performance, a graphical analysis of the prediction results was conducted. Figure 8 presents scatter plots of predicted versus experimental CO_2_ solubility values for the DBO-LGBM and GWO-BPNN base learners, as well as for the stacking ensemble model, on both the training and test sets. As shown in figure 8, nearly all data points lie tightly clustered around the 45° diagonal line, indicating strong predictive power. The DBO-LGBM model exhibits two pronounced outliers on the test set where the prediction errors are relatively large, whereas the GWO-BPNN model displays no significant prediction bias. By effectively integrating the predictions from both base learners, the stacking ensemble yields a set of points that is more tightly clustered and uniformly distributed along the diagonal, thereby achieving superior fitting accuracy and robustness.

**Figure 8.**
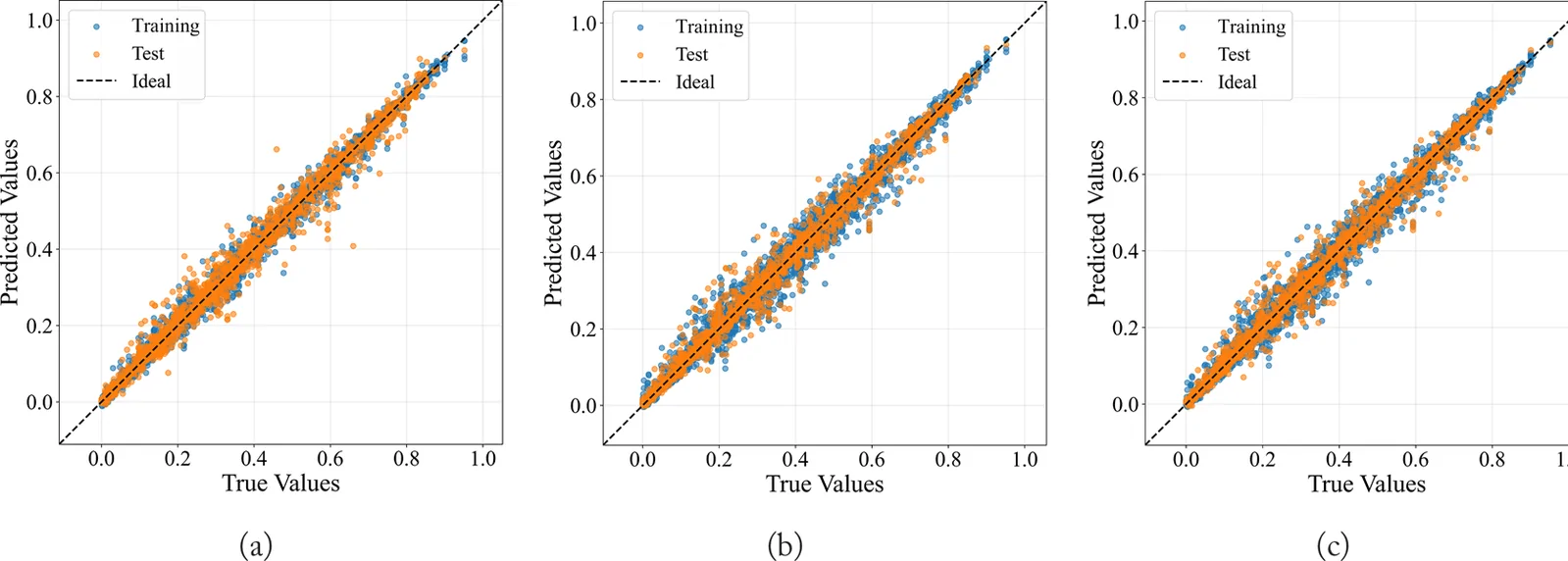
Prediction results scatter plot: (a) DBO-LGBM, (b) GWO-BPNN, and (c) stacking.

Figure 9 further illustrates the predictive performance of the DBO-LGBM and GWO-BPNN base learners, as well as the stacking ensemble model, using residual plots and error distribution histograms. The residual plots confirm that the stacking model's errors are evenly distributed around zero across both the training and test sets, with small fluctuations and no discernible trend relative to the actual values. This pattern indicates the absence of systematic bias, overfitting or underfitting, supporting the model's robustness and generalizability. The error distribution histograms further reveal that the prediction errors of all models approximately follow a normal distribution centred near zero, with the stacking model exhibiting the highest density around zero. The number of samples with prediction errors close to zero is greater for the stacking model than for the two base learners, indicating that the ensemble approach further reduces overall prediction error and enhances predictive accuracy.

**Figure 9.**
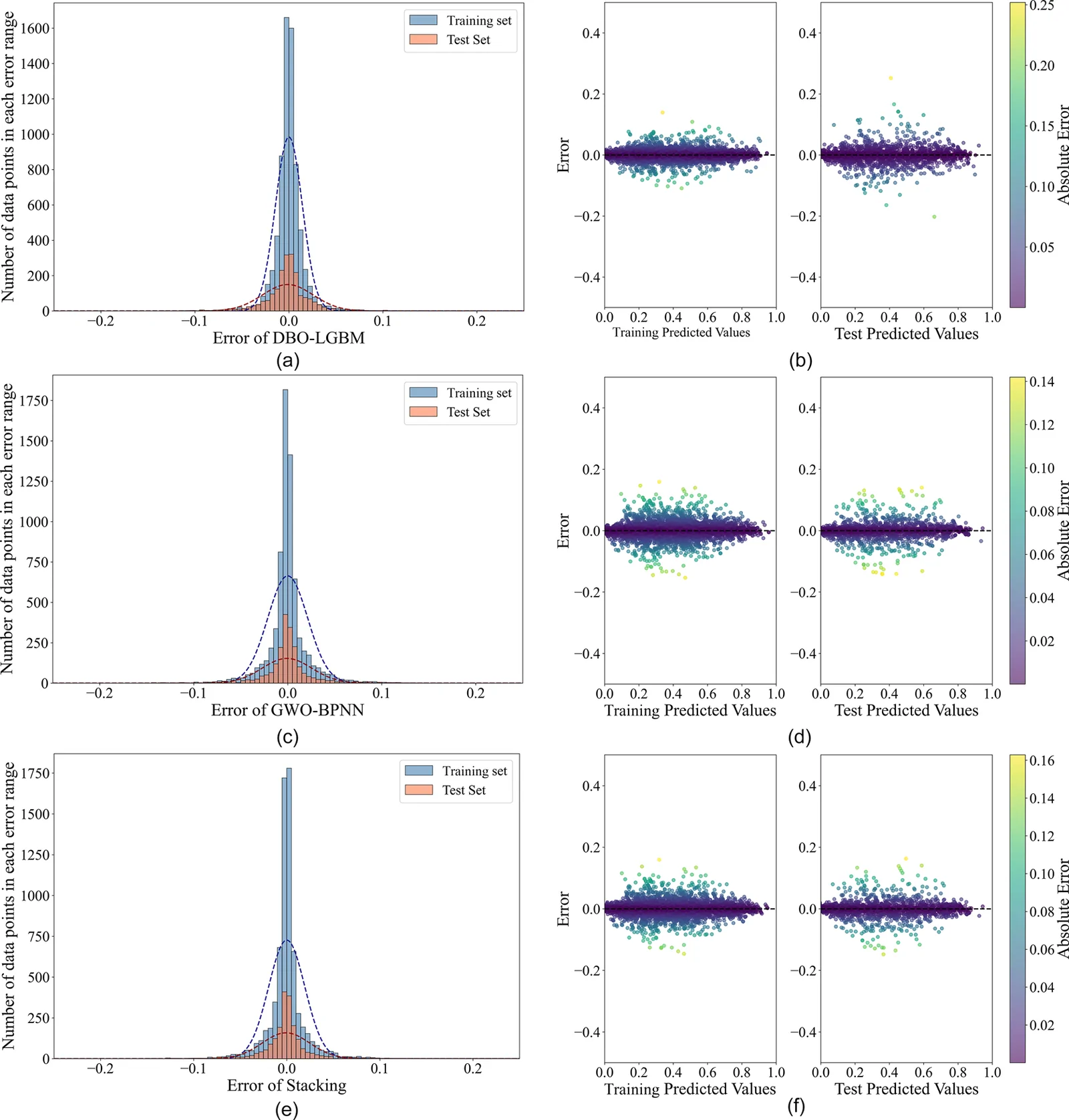
Error distribution histogram and residual plot: (a) error distribution histogram of DBO-LGBM, (b) residual plot of DBO-LGBM, (c) error distribution histogram of GWO-BPNN, (d) residual plot of GWO-BPNN, (e) error distribution histogram of stacking, and (f) residual plot of stacking.

### 3.2. Effect of data-splitting strategy on model performance

In this study, a random split strategy was initially adopted to construct the training, validation and test sets by randomly sampling data from the entire dataset for model training and performance evaluation. Under this strategy, the training and test sets may contain ILs with similar cation and anion structures measured under different experimental conditions. Therefore, the resulting test performance primarily reflects the model's ability to predict the behaviour of known IL systems across a range of temperatures and pressures. This data partitioning approach is widely used in data-driven modelling studies and is suitable for evaluating model performance within known chemical spaces. In contrast, Bastami *et al.* [33] employed a more stringent data partitioning strategy, in which certain IL structures were deliberately excluded from the training set and reserved exclusively for testing. This approach enables the assessment of a model's extrapolation capability towards previously unseen IL systems.

To further evaluate the generalization ability of the proposed model to new IL systems, we adopted a structure-based data-splitting strategy following the methodology of Bastami *et al.* Four cations ([C_8_F_13_MIM], [OMPYR], [OMIM] and [N_1,8,8,8_]) and three anions ([FSA], [DCA] and EtSO_4_) were deliberately withheld during the model development stage. All samples containing any of these selected ions were assigned exclusively to the test set, while the remaining samples were used to construct the training and validation sets. This splitting process ultimately resulted in 7678 samples for training, 1097 samples for validation and 1341 samples for testing. Since the IL structures in the test set were completely unseen during training, this partitioning strategy created an ‘unseen ionic liquid prediction’ scenario, providing a more realistic assessment of the model's ability to predict CO_2_ solubility in new IL systems.

As shown in table 8, the results show that models maintained relatively small differences between training and test performance metrics, indicating a certain degree of predictive capability. By combining the strengths of both LGBM and neural network models, the stacking ensemble achieved the best overall performance on both the training and test sets. Specifically, the test-set MAE, MSE and *R*^2^ reached 0.0360, 0.0028 and 0.9456, respectively, slightly outperforming the stand-alone DBO-LGBM and GWO-BPNN models. These results demonstrate the superior generalization capability and predictive robustness of the stacking framework, enabling accurate predictions for previously unseen ILs.

**Table 8.** Model performance under structural grouping data split.

	MAE		MSE		*R*^2^	
method	training	test	training	test	training	test
DBO-LGBM	0.0088	0.0349	0.0002	0.0029	0.9965	0.9439
GWO-BPNN	0.0116	0.0384	0.0004	0.0031	0.9927	0.9404
stacking	0.0104	0.0360	0.0003	0.0028	0.9941	0.9456

A comparison with the random split results further highlights the impact of the data partitioning strategy. Under the random split setting, all three models achieved very high predictive accuracy, with the stacking model obtaining a test-set *R*^2^ of 0.9888. However, when the IL structures in the test set were completely excluded from the training phase, the performance of all models decreased to varying degrees. For example, the test-set *R*^2^ of GWO-BPNN decreased from 0.9878 to 0.9404, whereas that of DBO-LGBM dropped from 0.9872 to 0.9439. The observed performance degradation indicates that predicting previously unseen ILs is substantially more challenging than predicting known systems under different operating conditions. Nevertheless, it is noteworthy that the stacking model still maintained a high *R*^2^ of 0.9456 and low prediction errors when applied to ILs that were completely absent from the training set. This suggests that the model has learned meaningful structure–property relationships governing CO_2_ solubility rather than merely relying on memorized information from previously observed samples. Overall, although predicting unseen ILs is a more challenging extrapolation task, the proposed stacking model still demonstrates excellent predictive performance, strong structural generalization and considerable practical value. These findings indicate that the model can serve as a reliable tool for screening and designing novel ILs for CO_2_ capture applications.

### 3.3. Effect of feature sets on model performance

To further evaluate the ability of Morgan fingerprints to represent the structural information of ILs and to assess the impact of incorporating additional molecular descriptors on predictive performance, a hybrid feature set was constructed by integrating physico-chemical property descriptors and charge-distribution descriptors into the Morgan fingerprint representation. The molecular descriptors were calculated using RDKit and comprised nine representative features, as listed in table 9: TPSA, MolWt, MolLogP, Maximum E-State Index, Minimum E-State Index, NumHAcceptors, NumHDonors, FractionCSP3 and HeavyAtomCount. These descriptors characterize ILs from multiple perspectives, including physico-chemical properties, electronic states and structural features, thereby providing more comprehensive molecular information for model development. In addition, charge-distribution descriptors were incorporated to capture the local electronic environment of ILs and their potential electrostatic interactions with CO_2_ molecules. The resulting feature set was then employed to develop the DBO-LGBM, GWO-BPNN and stacking models, and the corresponding predictive performance metrics are summarized in table 10.

**Table 9.** Molecular descriptors generated based on the RDKit.

molecular descriptors	descriptors
TPSA	topological Polar Surface Area
MolWt	Molecular Weight
MolLogP	Octanol-Water Partition Coefficient
MaxAbsEStateIndex	maximum E-state index
MinAbsEStateIndex	minimum E-state index
NumHAcceptors	Number of Hydrogen Bond Acceptors
NumHDonors	Number of Hydrogen Bond Donors
FractionCSP3	Fraction of sp³-Hybridized Carbons
HeavyAtomCount	Number of Heavy Atoms

**Table 10.** Model performance after incorporating additional molecular features.

	MAE		MSE		*R*^2^	
method	training	test	training	test	training	test
DBO-LGBM	0.0071	0.0164	0.0001	0.0007	0.9976	0.9873
GWO-BPNN	0.0130	0.0158	0.0005	0.0007	0.9915	0.9875
stacking	0.0108	0.0147	0.0003	0.0006	0.9939	0.9889

Compared with results obtained using only Morgan fingerprints, the inclusion of physico-chemical properties and charge-distribution descriptors yields only marginal changes in model performance. Specifically, for the DBO-LGBM model, the MAE on the test set slightly decreases from 0.0166 to 0.0164, while the *R*^2^ increases from 0.9872 to 0.9873. For the GWO-BPNN model, the MAE increases from 0.0154 to 0.0158, and the *R*^2^ decreases from 0.9878 to 0.9875. For the stacking model, the MAE decreases from 0.0149 to 0.0147 (approx. a 1.34% reduction), accompanied by a slight increase in *R*^2^ from 0.9888 to 0.9889. Overall, incorporating additional descriptors does not yield a noticeable improvement in predictive performance, indicating that the newly added features provide limited additional information for CO_2_ solubility prediction.

This result suggests that Morgan fingerprints can adequately encode the key structural information governing CO_2_ solubility. By capturing atomic neighbourhood environments and molecular topological connectivity, Morgan fingerprints effectively represent the structural characteristics of IL cations and anions, which largely determine system polarity, steric effects and intermolecular interaction capabilities. As shown in the previous results, models using only Morgan fingerprints achieved test *R*^2^ values exceeding 0.987, indicating that structural information alone is sufficient to explain most of the variation in CO_2_ solubility.

By contrast, the additional physico-chemical and charge-related descriptors may partially overlap with the information already embedded in Morgan fingerprints. For instance, molecular weight, TPSA, hydrogen-bond donor/acceptor counts and LogP are derived directly or indirectly from molecular structure, and their variations are inherently reflected in the topological features encoded by Morgan fingerprints. Consequently, when these descriptors are combined with Morgan fingerprints, the additional information provided to the models is limited, yielding negligible performance gains. Moreover, introducing additional descriptors increases the dimensionality of the feature space, whereas the amount of new, effective information does not increase proportionally, potentially introducing redundancy. This is also supported by the slight performance degradation observed in the GWO-BPNN model after adding extra descriptors, suggesting that simply increasing feature dimensionality does not necessarily improve predictive accuracy.

From a mechanistic perspective, the solubility of CO_2_ in ILs is primarily governed by molecular structural features. The interactions between CO_2_ and ILs involve electrostatic interactions, induction effects, van der Waals forces and weak hydrogen bonding, all of which are strongly dependent on functional group composition, atomic connectivity and local chemical environments. Therefore, Morgan fingerprints, which directly encode molecular topology, inherently provide strong representational power. In comparison, physico-chemical properties and charge-distribution descriptors can be regarded as derivative representations of structural information and thus provide limited additional insight.

In summary, for the present CO_2_ solubility prediction task, Morgan fingerprints already provide sufficiently rich and effective structural representations. Although the inclusion of physico-chemical and charge-related descriptors yields minor performance improvements in some cases, the gains are marginal relative to the increased feature dimensionality and model complexity. Therefore, considering predictive accuracy, model complexity and generalization capability, Morgan fingerprints alone are adequate for molecular representation in this study.

### 3.4. Multi-model comparison experiment

To further evaluate the performance advantage of the stacking ensemble model for CO_2_ solubility prediction, several mainstream regression models, including SVM, ANN, decision tree (DT) and RF, were reconstructed and independently trained based on the same CO_2_ solubility dataset developed in this study. All models were trained and tested under identical data-splitting settings and feature inputs and evaluated using the same performance metrics to ensure a fair and consistent comparison. The results are shown in figure 10. The results indicate that the stacking model achieves the highest *R*^2^ and the lowest MSE and MAE, indicating superior prediction accuracy and stability compared with traditional single models. Combined with the results in §3.1, LGBM and BPNN both exhibit higher test-set *R*^2^ values than SVM, ANN, DT and RF, further validating the rationale for selecting LGBM and BPNN as base learners for the stacking ensemble and providing a solid basis for the ensemble model's excellent performance.

**Figure 10.**
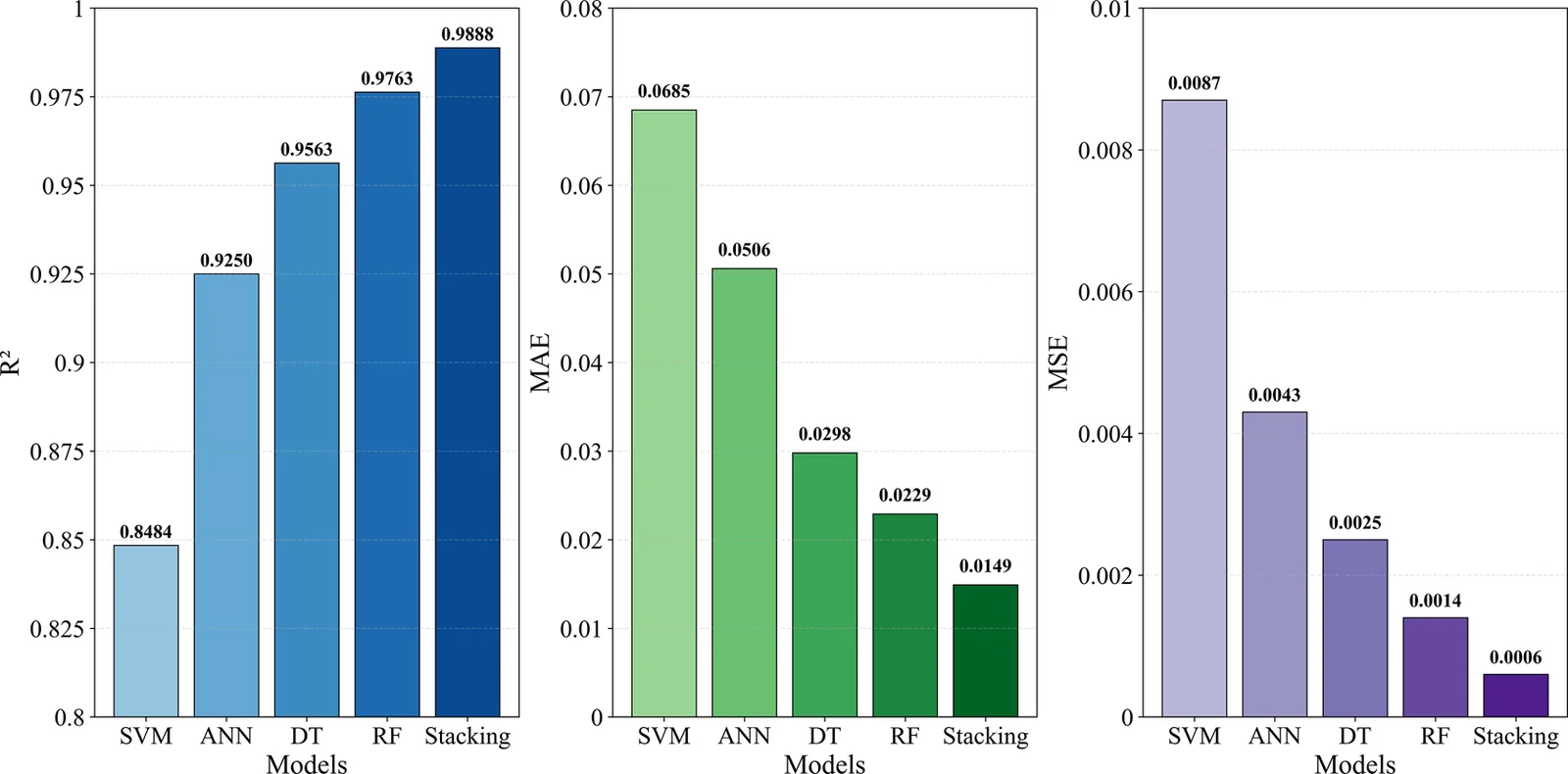
Multi-model comparison results.

To comprehensively evaluate the performance of the proposed method for CO_2_ solubility prediction and to compare it with the latest advances in the field, four representative machine learning models from recent years were further introduced [34], including deep neural networks (DNN), Bayesian neural networks (BNN), TabNet and GrowNet. Among them, DNN serves as a classical representative of deep learning models, while BNN, TabNet and GrowNet, respectively, represent recent advances in Bayesian learning, attention-driven tabular data modelling and progressive ensemble learning frameworks, thereby ensuring the comprehensiveness and fairness of the comparison. All models were trained and evaluated under identical experimental settings, and the hyperparameters of each model were optimized using the grid search strategy reported in the literature.

A clear trade-off between computational efficiency and predictive performance is observed across all models. The results are shown in table 11. Although DNN achieves relatively fast training (13.52 s), its predictive accuracy remains limited (*R*^2^ = 0.9859). By contrast, BNN and TabNet improve model expressiveness but incur substantial computational costs (658.47 and 183.80 s, respectively), indicating that more complex models do not necessarily yield proportional performance gains in this task. Meanwhile, GrowNet incurs a low computational cost (26.72 s) but achieves lower accuracy (*R*^2^ = 0.9299), suggesting that lightweight ensemble learning alone is insufficient for high fidelity prediction.

**Table 11.** MAE, MSE and *R*^2^ of various models on training and test data.

	MAE		MSE		*R*^2^		time (s)
method	training	test	training	test	training	test	
DNN	0.0167	0.0180	0.0007	0.0008	0.9880	0.9859	13.52
BNN	0.0343	0.0355	0.0020	0.0021	0.9647	0.9626	658.47
TabNet	0.0335	0.0331	0.0021	0.0020	0.9620	0.9644	183.8
GrowNet	0.0518	0.0536	0.0037	0.0040	0.9327	0.9299	26.72
stacking	0.0112	0.0149	0.0003	0.0006	0.9932	0.9888	75.91

In comparison, the proposed stacking model achieves the best overall performance with a moderate computational cost (75.91 s). It effectively integrates complementary strengths of the base learners, improving generalization while avoiding the inefficiencies of complex models (BNN and TabNet) and the accuracy limitations of lightweight models (DNN and GrowNet), thereby achieving an optimal balance between accuracy and efficiency.

From an interpretability perspective, the proposed tree-based ensemble model provides intrinsic feature interpretability, enabling direct association between feature contributions and chemically meaningful descriptors such as molecular structure and electronic effects. By contrast, although DNN and BNN can be interpreted using SHAP-based analyses, their highly nonlinear architectures limit direct mapping between feature importance and explicit chemical mechanisms, resulting in weaker structure–property interpretability.

### 3.5. Optimization method comparison experiment

To evaluate the effectiveness of the selected hyperparameter optimization methods, particle swarm optimization (PSO) and artificial hummingbird algorithm (AHA) are additionally employed to optimize the LGBM and BPNN models, allowing a comparison of the performance improvements achieved by different swarm intelligence strategies. Figure 11a,b displays the hyperparameter optimization curves obtained with the various algorithms. During the optimization of LGBM hyperparameters, the DBO algorithm attains the best predictive performance on the validation set, reaching an *R*^2^ of 0.9903 after 30 iterations and substantially enhancing the model. By contrast, the PSO optimization process exhibits relatively large fluctuations, indicating greater uncertainty and lower stability in hyperparameter tuning compared with DBO and AHA. Although AHA shows a relatively smooth convergence profile, its final optimized performance does not surpass that achieved by DBO. For BPNN hyperparameter optimization, GWO demonstrates the fastest convergence rate and the highest optimization efficiency. Early in the iterative process, the GWO curve rises sharply and stabilizes at an *R*^2^ of 0.9896, effectively circumventing local optima. These experimental results confirm the rationality and effectiveness of selecting DBO and GWO as the optimization algorithms in this study.

**Figure 11.**
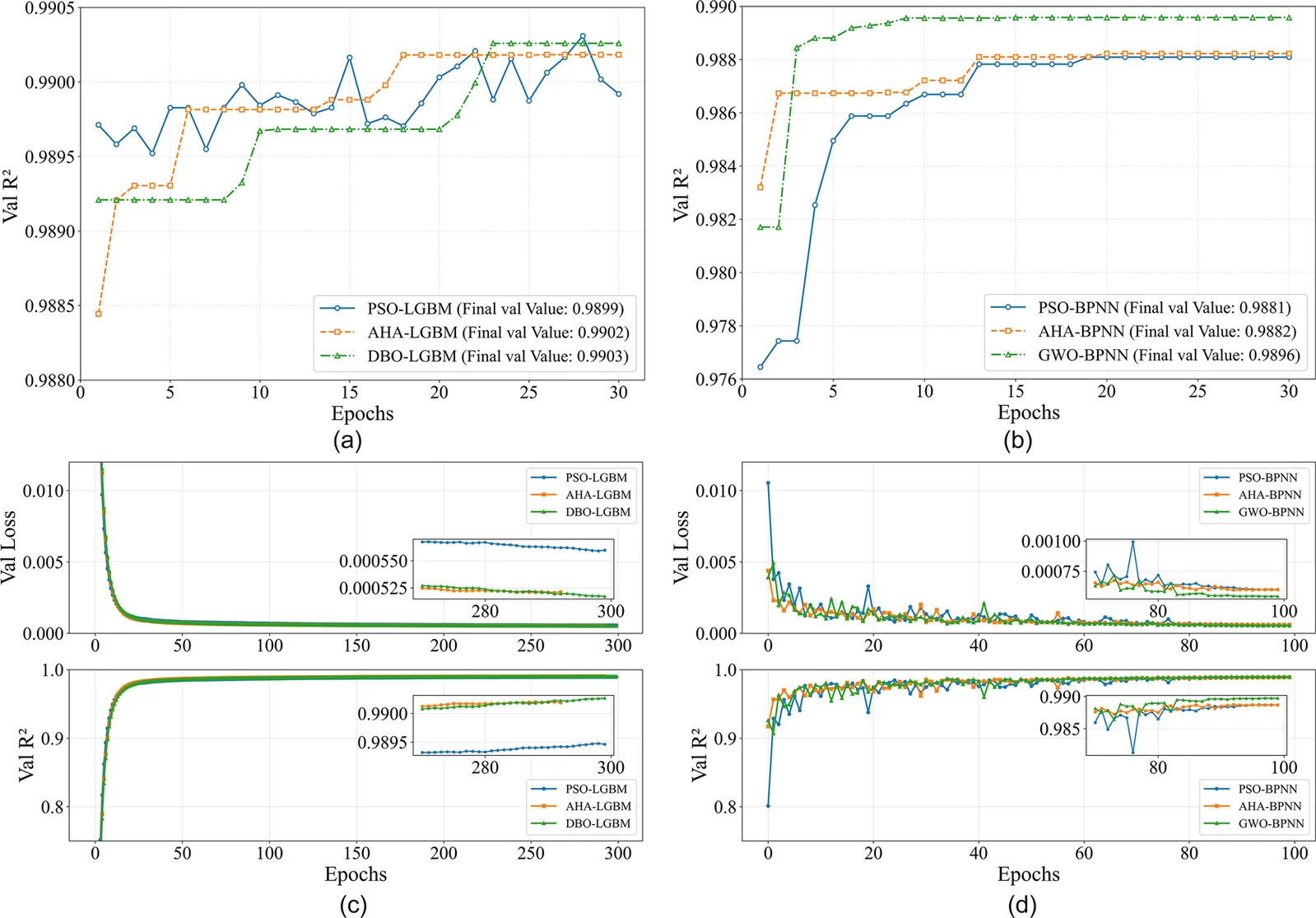
Optimization comparison curves, validation loss curve and *R*^2^ change curves: (a) optimization comparison curves for LGBM, (b) optimization comparison curves for BPNN, (c) validation loss curves and *R*^2^ change curves for LGBM, and (d) validation loss curve and *R*^2^ change curves for BPNN.

Figure 11c,d indicates that, compared with PSO and AHA, the *R*^2^ curves of DBO and GWO converge more smoothly and ultimately attain higher accuracy. The loss curves of DBO and GWO decrease more rapidly and stably, reaching early convergence and then maintaining a steady state. Notably, for BPNN, the fluctuations of GWO in later iterations are markedly smaller than those of PSO and AHA, reflecting stronger fitting capability and convergence performance. These findings confirm that DBO and GWO effectively avoid severe oscillations and escape local optima during hyperparameter optimization, yielding superior model performance with higher prediction accuracy and fitting quality compared with the other algorithms. Table 12 reports the MAE, MSE and *R*^2^ values obtained by the four optimization algorithms on both the training and test sets. On the test set, DBO-LGBM and GWO-BPNN again demonstrate superior performance.

**Table 12.** MAE, MSE and *R*^2^ for each model's training and test sets.

	MAE		MSE		*R*^2^	
method	training	test	training	test	training	test
AHA-LGBM	0.0091	0.0169	0.0002	0.0008	0.9963	0.9867
PSO-LGBM	0.0092	0.0167	0.0002	0.0008	0.9961	0.9866
DBO-LGBM	0.0091	0.0166	0.0002	0.0007	0.9963	0.9872
AHA-BPNN	0.0153	0.0176	0.0006	0.0008	0.9898	0.9858
PSO-BPNN	0.0181	0.0201	0.0007	0.0009	0.9874	0.9837
GWO-BPNN	0.0122	0.0154	0.0004	0.0007	0.9921	0.9878

By integrating tree-based and neural network algorithms, the stacking ensemble model significantly enhances model diversity and representational capacity. This architecture constitutes a robust and reliable framework, consistently delivering strong performance on both the training and test sets. These results strengthen confidence in the model's ability to generalize effectively to unseen data.

### 3.6. Model interpretability analysis

After analysing the models’ predictive performance, SHAP is employed to investigate the key factors influencing CO_2_ solubility predictions. SHAP [35] is an advanced interpretability tool for explaining machine learning model predictions. Based on the concept of Shapley values from cooperative game theory, SHAP assigns each input feature a quantitative contribution to the model prediction. The SHAP value represents the contribution of each feature to a given sample.

This method focuses on individual sample predictions, assigning an importance value to each feature to explain the model output. It not only quantifies each feature's influence on the prediction but also clearly indicates whether the effect is positive or negative, thereby revealing the specific contributions of different substructures to CO_2_ solubility. Since tree-based models generally exhibit superior interpretability, the DBO-LGBM model is selected for SHAP analysis.

#### 3.6.1. Key driving factors for CO_2_ solubility

Figure 12a displays the SHAP feature importance beeswarm plot. In this visualization, features are ranked vertically by importance, with the most influential appearing at the top. Each point represents an individual sample and is positioned horizontally according to its SHAP value, which quantifies the magnitude of that feature's impact on the model's prediction for that instance. Points located to the right of the centreline correspond to positive SHAP values, indicating features that increase the predicted output, while points to the left indicate negative contributions that decrease the output.

**Figure 12.**
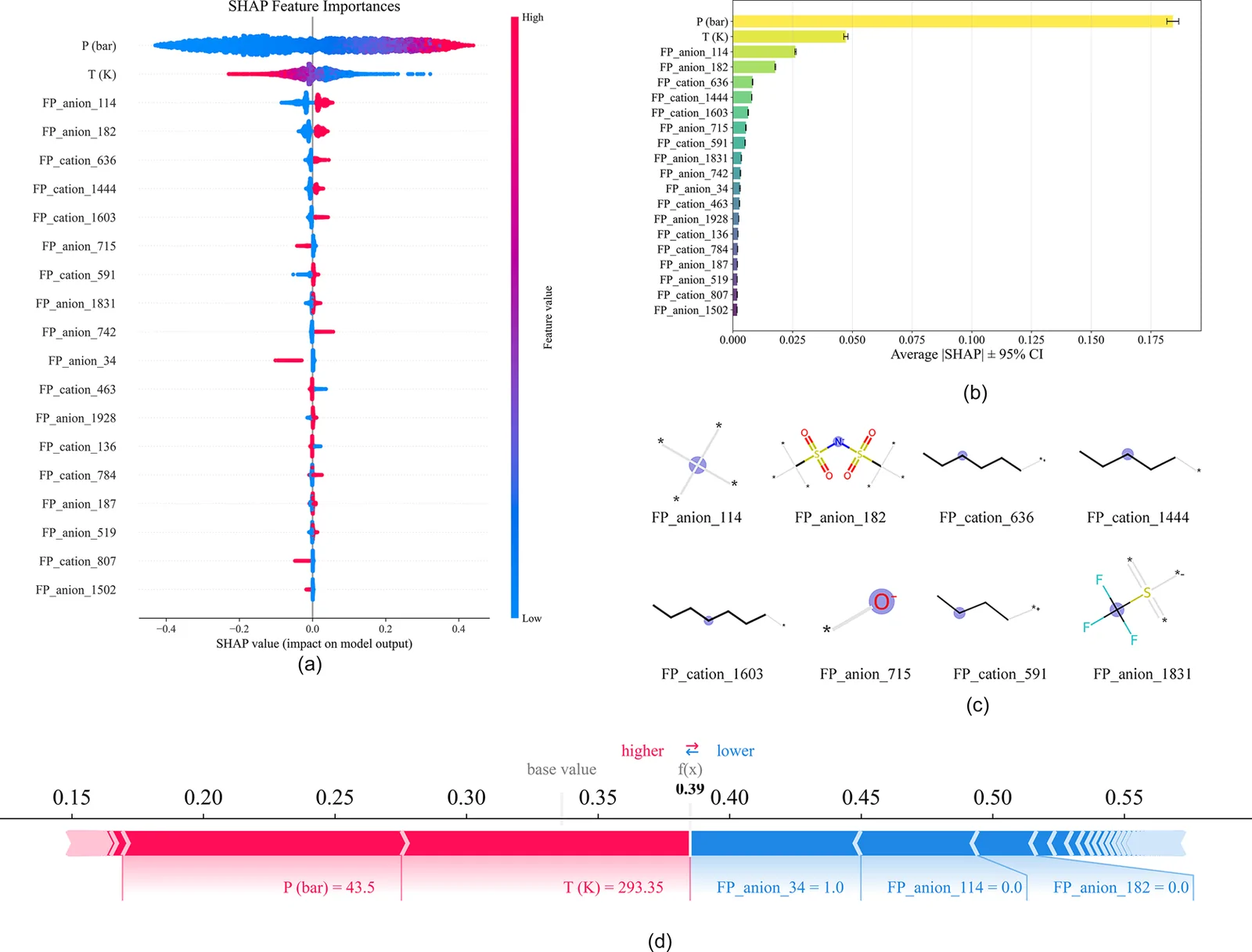
SHAP interpretability analysis: (a) feature importance beeswarm plot, (b) feature importance ranking with confidence intervals, (c) visualization of the most important Morgan fingerprint substructures, and (d) SHAP force plot example for CO_2_ solubility prediction.

As shown in figure 12a, the two most important features are the experimental conditions—pressure (*P*) and temperature (*T*)—which also exhibit the widest SHAP value ranges, indicating that they have the most significant impact on the model's predictions. An increase in pressure has a positive effect on the model output. High pressure is beneficial for increasing the model-predicted CO_2_ solubility in ILs. From a thermodynamic perspective, the solubility of CO_2_ in ILs is fundamentally governed by gas–liquid phase equilibrium. According to Henry's law, higher pressure increases the chemical potential of CO_2_ in the gas phase, thereby enhancing the driving force for equilibrium between the gas and liquid phases, promoting the transfer of CO_2_ into the liquid phase and increasing its absorption capacity. By contrast, an increase in temperature negatively affects the model output. Most physical absorption processes of CO_2_ in ILs are exothermic (ΔH_sol < 0); thus, elevated temperatures shift the absorption equilibrium towards desorption and simultaneously increase the Henry constant, reducing CO_2_ solubility in the IL. Therefore, the importance of pressure (*P*) and temperature (*T*) not only reflects the model's sensitivity to experimental conditions but also aligns with the fundamental thermodynamic principles governing CO_2_ absorption.

Regarding the IL substructures, FP_anion_114 ranks first in importance. Fingerprint mapping analysis indicates that this site, along with FP_anion_182 and FP_anion_1831, corresponds to local structural features of the [Tf_2_N] anion. The [Tf_2_N] anion possesses a large molecular volume and a highly delocalized negative charge distributed over the N(SO_2_CF_3_)_2_ framework, resulting in reduced local charge density. From a quantum-chemical perspective, charge delocalization weakens coulombic interactions between cations and anions. The weaker ion pairing increases the free volume and structural flexibility of the IL, thereby providing more available solvation space for CO_2_ molecules and significantly enhancing its physical solubility. Moreover, the –-CF_3_ groups in [Tf_2_N] are highly polarizable, thereby strengthening van der Waals interactions with CO_2_. DFT studies have further shown that the electron-deficient carbon atom in CO_2_ can engage in weak electrostatic, quadrupole and induced-dipole interactions with fluorine atoms and sulfonyl oxygens in [Tf_2_N], thereby reducing the solvation free energy of CO_2_ and enhancing its solubility. By contrast, smaller anions, such as [Cl] and [BF_4_], tend to form tighter ion pairs with cations, reducing free volume and hindering CO_2_ solubility. In addition, [Tf_2_N]-based ILs typically exhibit lower viscosity, thereby facilitating CO_2_ diffusion and mass transfer and resulting in superior absorption performance.

Among cationic features, FP_cation_636 has the highest importance, corresponding, together with FP_cation_1444, FP_cation_1603 and FP_cation_159, to the local structural features of long-chain imidazolium cations. Compared with short alkyl chain cations such as 1-ethyl-3-methylimidazolium ([EMIM]) and 1-butyl-3-methylimidazolium ([BMIM]), longer alkyl chains increase structural asymmetry and weaken cation–anion interactions, thereby enlarging the system free volume and generating additional nanoscale cavities for CO_2_ solvation. MD studies further indicate that long-chain imidazolium ILs tend to form nanophase-segregated structures with coexisting polar and non-polar domains, where CO_2_ preferentially partitions into the non-polar regions formed by the alkyl chains. Consequently, the non-polar microenvironments created by long alkyl chains provide favourable solvation sites for CO_2_, enhancing its solubility. Additionally, the high polarizability of the imidazolium ring and its π-electron system can interact with CO_2_ through weak quadrupole-induced dipole interactions, further stabilizing solvated CO_2_.

Therefore, combining the [Tf_2_N] anion with imidazolium cations bearing longer alkyl chains emerges as an ideal strategy for constructing high-performance CO_2_-absorbing ILs.

FP_anion_715 corresponds to oxygen-based anions, which are substructures of anions such as [MeSO_3_] and [HSO_4_], and exert a negative effect on CO_2_ absorption. At the microscopic level, oxygen atoms exhibit high local charge density, thereby strengthening electrostatic interactions and hydrogen-bond networks with cations. Quantum-chemical studies suggest that increased local charge density enhances cation–anion electrostatic interactions and ion association, thereby increasing the system's cohesive energy. Strong ion pairing and hydrogen-bond networks result in a denser IL structure, reducing free volume and increasing viscosity. The reduction in free volume limits CO_2_ solubility and diffusion, while higher viscosity further impedes mass transfer. Moreover, although these anions contain oxygen atoms, their overall basicity is relatively low, rendering them incapable of forming highly efficient and reversible chemical interactions with CO_2_ and thus failing to compensate for their limited physical absorption capacity, ultimately leading to a negative contribution to CO_2_ uptake.

Overall, the key features identified by SHAP are consistent with the CO_2_ absorption mechanisms revealed by quantum-chemical and molecular-thermodynamic studies. High-performing ILs generally exhibit more pronounced charge delocalization, weaker ion pairing and more favourable CO_2_–IL intermolecular interactions. Thermodynamically, these characteristics manifest as lower solvation free energy and larger free volume. By contrast, high-charge-density anions form strong ion pairs and hydrogen-bond networks, increasing cohesive energy and reducing structural flexibility, which inhibits CO_2_ absorption. These results indicate that the developed model not only demonstrates robust predictive performance but also provides mechanistic insights into the structure–property relationships of ILs, including electronic structure, intermolecular interactions and thermodynamic principles.

Figure 12b presents the feature importance ranking with confidence intervals. The horizontal lines next to each bar represent the 95% confidence interval of the feature importance estimate. Narrower confidence intervals indicate more precise and consistent estimates of feature importance across different training runs. The top 20 most important features all exhibit relatively narrow confidence intervals, demonstrating high stability in their contributions to the model. The important Morgan fingerprint substructures identified by SHAP are visualized in figure 12c.

#### 3.6.2. Robustness analysis of [tf_2_N] feature importance via downsampling

We observed that the number of IL samples containing [Tf_2_N] in the dataset was 4301, substantially higher than that for other anion categories. Such data imbalance may cause the model to become biased towards majority classes during training, thereby reducing its ability to generalize to minority classes. Therefore, a random downsampling strategy was applied to the samples containing [Tf_2_N].

First, all samples containing the [Tf_2_N] anion were separated from the dataset, while samples with other anions were retained as another subset. Let the total number of non-[Tf_2_N] samples be denoted as *N*_other_, and the number of non-[Tf_2_N] anion categories be denoted as *K*_other_. The target number of retained [Tf_2_N] samples was defined as follows:

(3.1) $$N_{target}=\left\lfloor \frac{N_{other}}{K_{other}}\right\rfloor ,$$

where ⌊ · ⌋ denotes the floor operation.

Subsequently, *N*_target_ [Tf_2_N]-containing samples were randomly selected from the original [Tf_2_N] subset without replacement. The retained [Tf_2_N] samples were then merged with all other anion samples to construct a newly balanced dataset, ensuring that the number of [Tf_2_N] samples was comparable to the average level of other anion categories. Finally, the resulting dataset was randomly shuffled to eliminate any potential ordering effects. After downsampling, the dataset size was reduced to 6030 samples. The dataset was then re-split, and the model was retrained for SHAP analysis. Downsampled feature importance beeswarm plot and feature importance ranking with confidence intervals are shown in figure 13.

**Figure 13.**
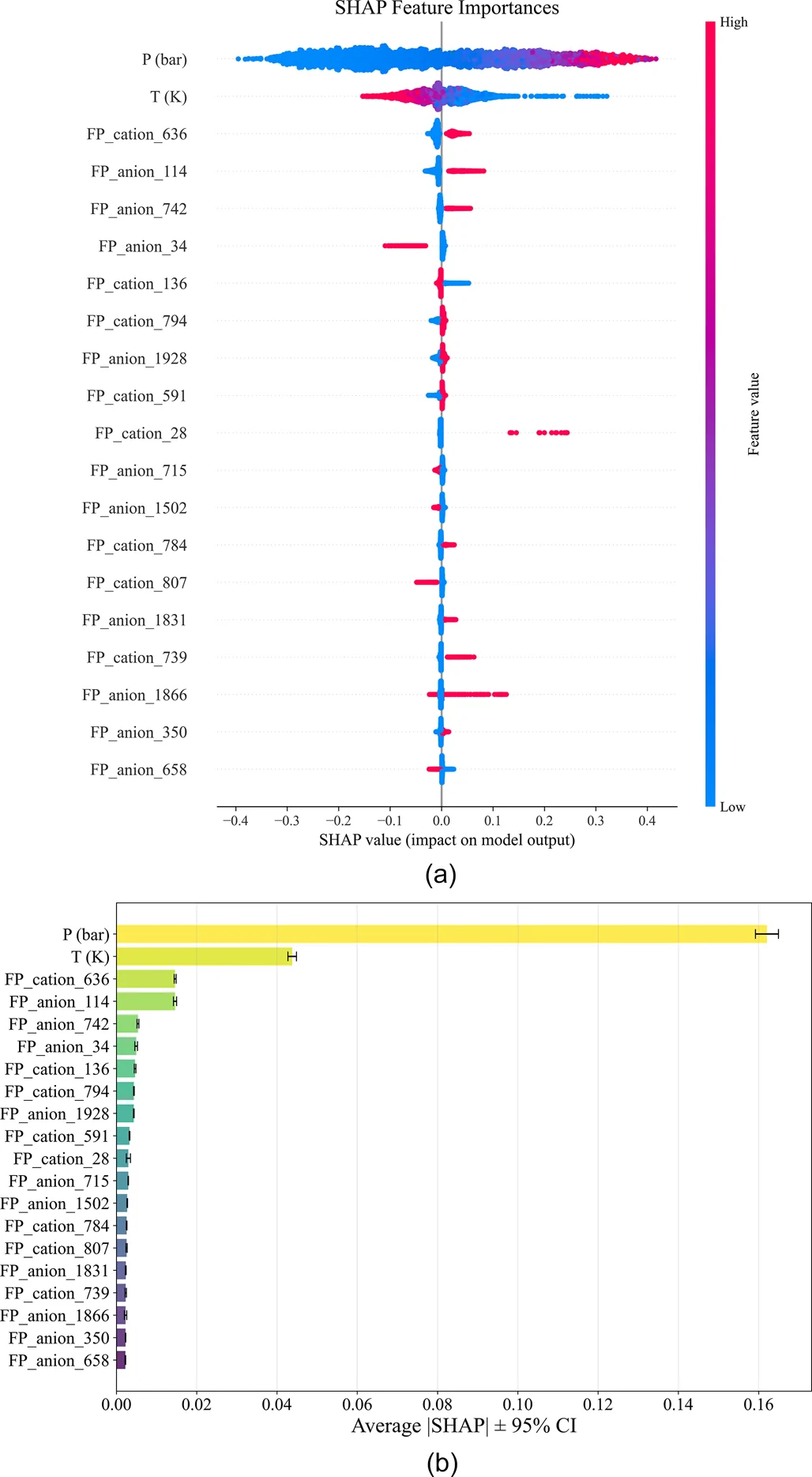
(a) Downsampled feature importance beeswarm plot and (b) feature importance ranking with confidence intervals based on downsampling.

The results show that the mean absolute SHAP value of FP_anion_114 decreased from 0.0262 to 0.0146, representing a reduction of approximately 44.3%, while FP_anion_1831 decreased from 0.0035 to 0.0023, corresponding to a reduction of approximately 34.3%. In addition, the feature importance ranking of FP_anion_114 changed from 1st to 2nd, whereas FP_anion_1831 dropped from 8th to 16th. These results indicate that the dominance of [Tf_2_N] in the sample indeed amplifies the importance of certain related features, demonstrating that data imbalance can influence SHAP interpretability to some extent. However, after undersampling, FP_anion_114 still maintained a relatively high importance and remained among the most influential features, suggesting that its contribution is not solely driven by the over-representation of [Tf_2_N] samples but is also closely related to the intrinsic effect of this anion on CO_2_ solubility. By contrast, FP_anion_1831 exhibited a more pronounced decrease in both importance and ranking, indicating that its contribution is influenced by both structural and sample-frequency effects. These results suggest that different [Tf_2_N]-related fingerprint features are affected to varying degrees by data imbalance.

Furthermore, as shown in table 13, the model performance changed only slightly after downsampling, indicating that the original model did not rely heavily on [Tf_2_N] samples for prediction and maintained good predictive stability. This suggests that the learned structure–property relationships are not solely driven by the over-representation of [Tf_2_N] but rather reflect, to some extent, the intrinsic contribution of anion structure to CO_2_ solubility.

**Table 13.** MAE, MSE and *R*^2^ for original and downsampled datasets’ training and test sets.

	MAE		MSE		*R*^2^	
method	training	test	training	test	training	test
original	0.0112	0.0149	0.0003	0.0006	0.9932	0.9888
downsampling	0.0112	0.0149	0.0004	0.0007	0.9933	0.9883

In summary, the downsampling experiment demonstrates that the sample dominance of [Tf_2_N] can enhance the importance of certain related features but does not alter the primary structure–property relationships identified by the model. These results validate the robustness of the SHAP analysis and further indicate that the importance of [Tf_2_N]-related features arises from a combined effect of structural contribution and sample frequency.

#### 3.6.3. SHAP force plot of CO_2_ solubility

Figure 12d shows an example of a CO_2_ solubility prediction using a SHAP force plot, which intuitively illustrates the model's decision process based on additive feature attribution. This plot clearly displays the contribution values, their positive or negative directions and the predicted solubility value for each feature in the selected sample. The *x*-axis represents SHAP values; red arrows indicate features that increase the prediction, while blue arrows indicate features that decrease it. The length of each arrow corresponds to the feature's weight, reflecting the magnitude of its influence. The final prediction is obtained by summing the baseline value and all SHAP values.

As shown, pressure and temperature (*P* = 43.5 bar, *T* = 293.35 K) have the greatest influence, and both increase the predicted value, further confirming that high pressure and low temperature enhance CO_2_ solubility. The substructures FP_anion_114 = 0, FP_anion_182 = 0 and FP_anion_34 = 1 all exert negative effects on the prediction. FP_anion_114 and FP_anion_182 correspond to [Tf_2_N] substructures; a value of zero indicates their absence, leading to reduced CO_2_ solubility. FP_anion_34 represents sulfur anions, a substructure of [SCN]. The presence of [SCN] reduces CO_2_ solubility owing to increased viscosity and restricted CO_2_ diffusion, as well as its strong polarity, which promotes ionic self-assembly into stable structures, thereby limiting CO_2_ dissolution. The baseline value is 0.336, and the final predicted value is 0.39, indicating relatively high CO_2_ solubility under the given conditions. The positive effects of high pressure and low temperature outweigh the negative effects of [SCN] and the absence of [Tf_2_N], resulting in an overall increase from the baseline.

#### 3.6.4. Interaction analysis of CO_2_ solubility

Figure 14 displays SHAP dependence plots for four key features governing CO_2_ solubility in ILs: temperature (*T*), pressure (*P*), FP_anion_114 and FP_cation_636. These plots illustrate how variations in feature values influence the predicted CO_2_ solubility. The *x*-axis represents the feature values, and the *y*-axis represents the corresponding SHAP values. Each point reflects the impact of a specific feature value on the predicted solubility.

**Figure 14.**
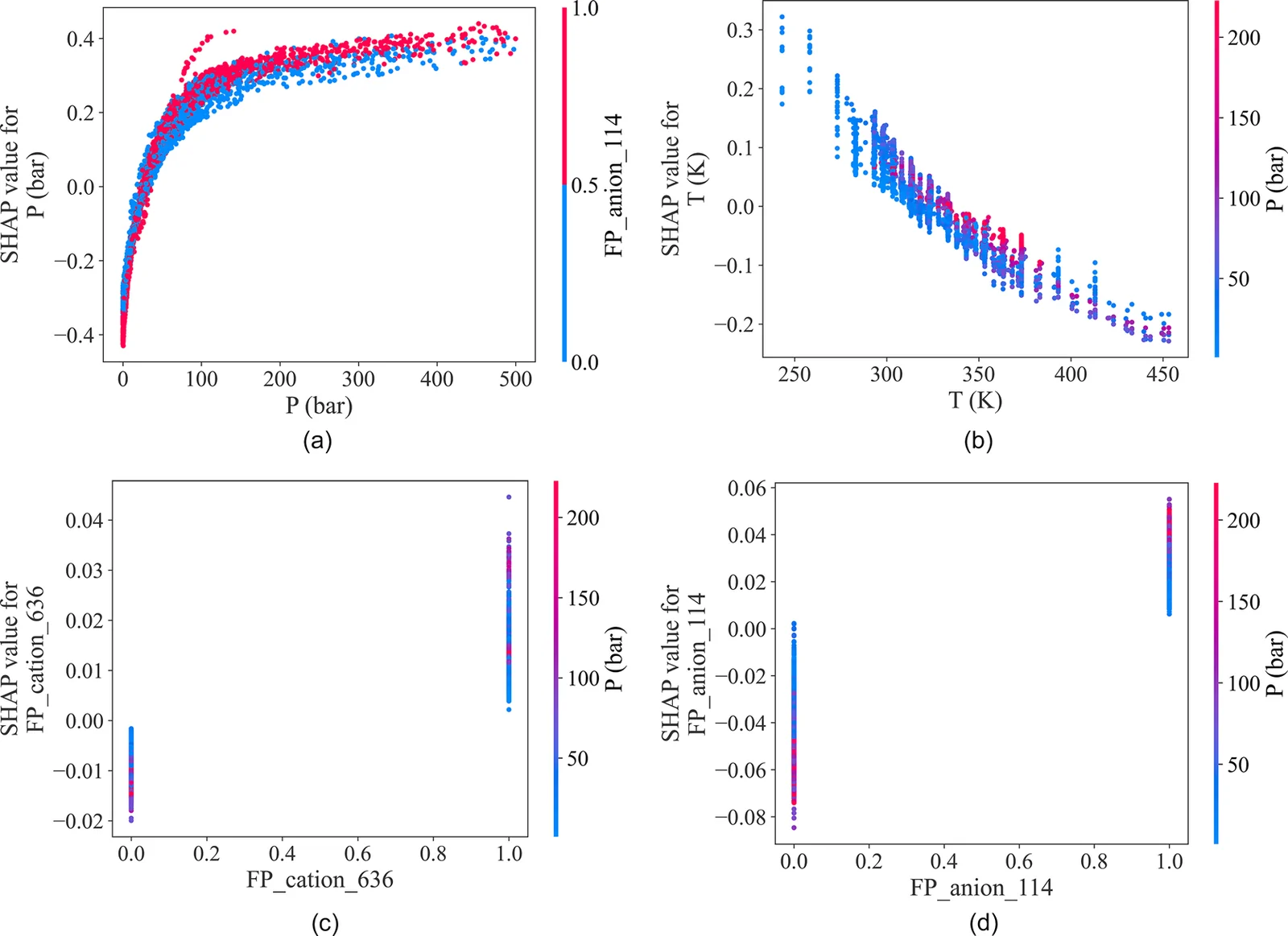
SHAP dependence plot: (a) *P*, (b) *T*, (c) FP_cation_636, and (d) FP_anion_114.

Figure 14a shows that SHAP values are negative at low pressure and gradually become positive as pressure increases, indicating that higher pressure promotes CO_2_ dissolution. Interaction with FP_anion_114 reveals that, at the same pressure, the presence of FP_anion_114 leads to higher SHAP values than its absence, further confirming its positive role in enhancing CO_2_ solubility. Figure 14b shows that SHAP values shift from positive to negative as temperature increases, indicating that CO_2_ solubility decreases with temperature. Interaction with pressure shows that higher pressure can mitigate the negative effect of elevated temperature. Figure 14c,d shows that when FP_anion_114 and FP_cation_636 are present (feature value = 1), SHAP values are positive, whereas their absence (feature value = 0) yields negative SHAP values, indicating that the presence of these substructures increases CO_2_ solubility.

#### 3.6.5. Correlation heat map analysis

Following the identification and analysis of key substructures, a correlation matrix of the Morgan fingerprints was computed to assess inter-feature relationships. High correlation coefficients indicate strong associations between fingerprint features. As shown in the heat map in figure 15, most regions appear in blue, reflecting generally low or even negative correlations among the molecular fingerprints. This outcome is desirable because it suggests that the selected fingerprints possess high diversity. Low inter-feature correlation means that they contribute more complementary information, enabling the model to capture patterns from multiple perspectives and thereby improving its generalization capability.

**Figure 15.**
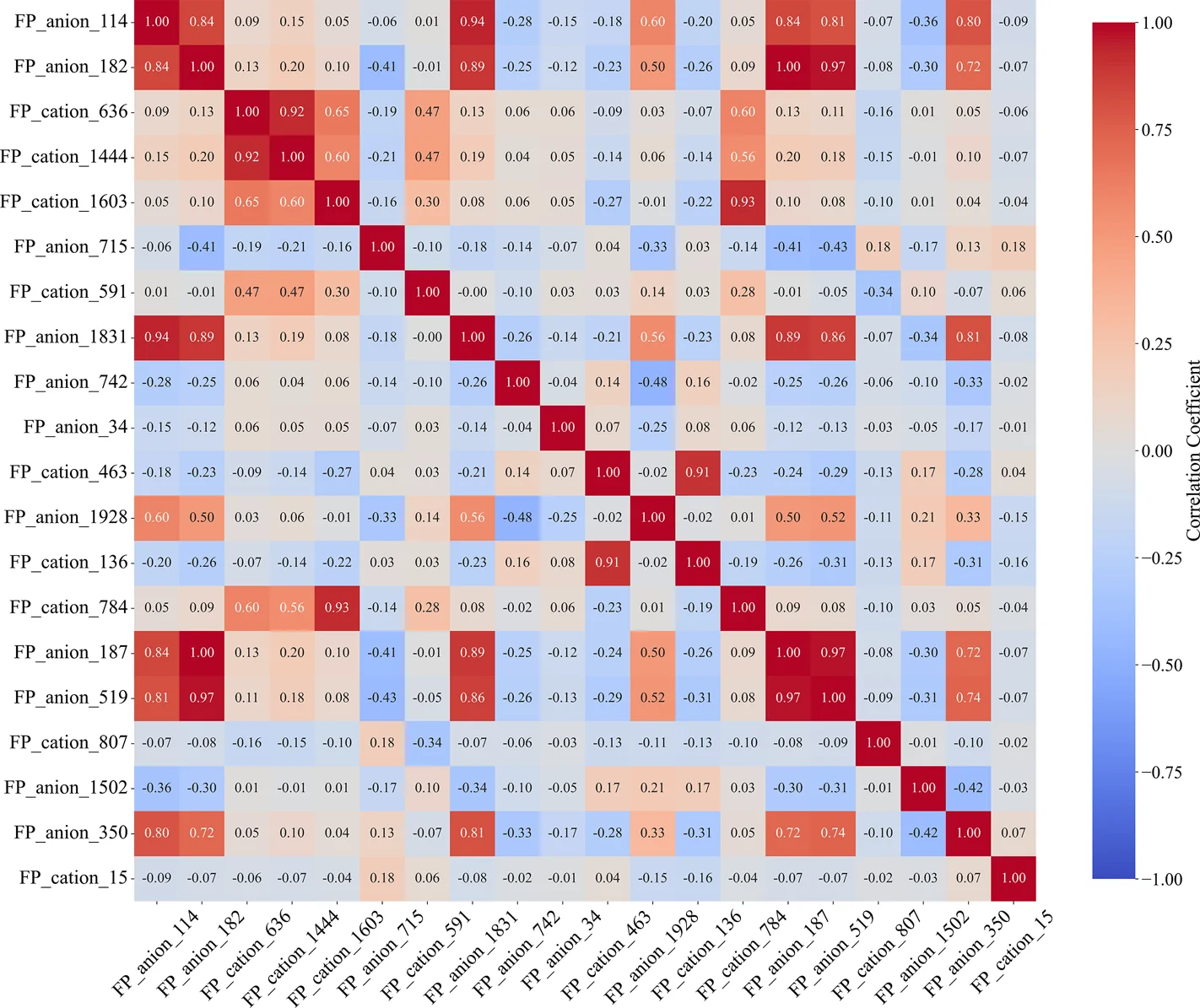
Heat map of molecular substructure correlation analysis.

### 3.7. Experimental validation

To verify the reliability of the proposed model for IL design, a closed-loop workflow comprising ‘model interpretation-structure screening-experimental validation’ was employed. First, key structural features governing CO_2_ adsorption performance were identified based on SHAP analysis results. In the anion category, the [Tf_2_N] anion, which exhibited a high SHAP contribution, was selected. For cations, a series of imidazolium-based cations with varying alkyl chain lengths were chosen, including [EMIM], [BMIM], 1-hexyl-3-methylimidazolium ([HMIM]), 1-octyl-3-methylimidazolium ([OMIM]) and 1-decyl-3-methylimidazolium ([DMIM]), to construct a set of target ILs for experimental CO_2_ adsorption validation.

The experiments were performed at 298.15 K and 0.1 MPa, and the results are presented in figure 16. The experimentally determined CO_2_ uptake followed the order: [DMIM][Tf_2_N] > [OMIM][Tf_2_N] > [HMIM][Tf_2_N] > [BMIM][Tf_2_N] > [EMIM][TF_2_N], which is in full agreement with the model-predicted trend. With increasing cation alkyl chain length, CO_2_ adsorption capacity shows a continuous increase, indicating that the model accurately captures the influence of structural variations on CO_2_ adsorption behaviour.

**Figure 16.**
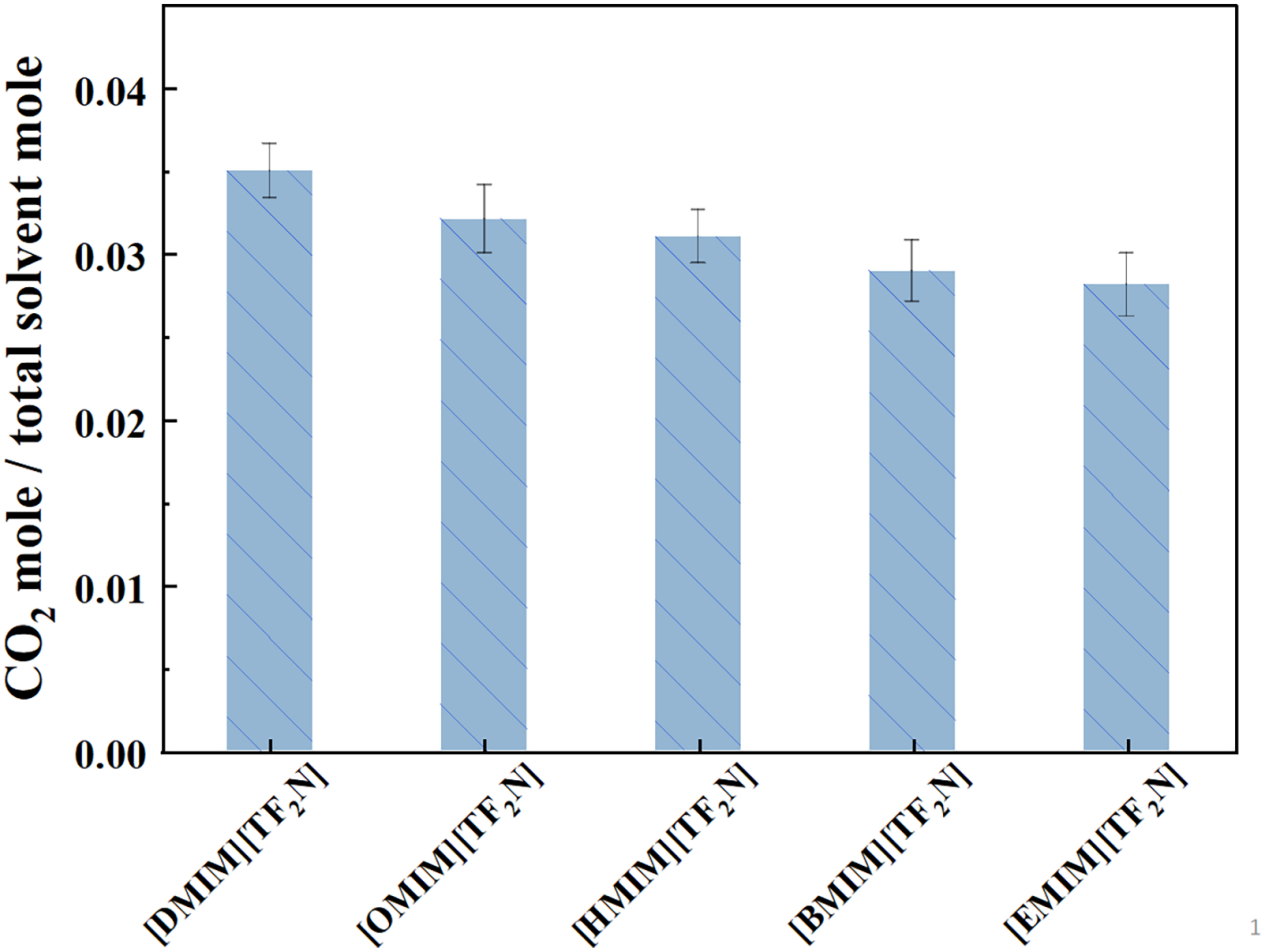
CO_2_ absorption capacity in [DMIM][TF_2_N], [OMIM][TF_2_N], [HMIM][TF_2_N], [BMIM][TF_2_N] and [EMIM][TF_2_N].

To further evaluate predictive accuracy, a quantitative comparison between experimental measurements and model predictions was performed, as summarized in table 14. The results show a strong linear correlation between predicted and experimental values, with a mean relative error of 5.14%. Among them, the model-predicted CO_2_ mole fraction in [DMIM][Tf_2_N] is 0.037, while the experimentally measured value is 0.035; for [EMIM][Tf_2_N], the predicted and experimental values are 0.026 and 0.028, respectively. The prediction deviations for the remaining samples remain within a small range, while [HMIM][Tf_2_N] shows excellent agreement between the predicted and experimental values. These results indicate that the model can not only accurately predict the relative performance ranking of different ILs but also reasonably estimate their actual adsorption capacities.

**Table 14.** Comparison of predicted and experimental CO_2_ absorption capacities for different ionic liquids.

IL	prediction	experiment
[DMIM][TF_2_N]	0.037	0.035
[OMIM][TF_2_N]	0.035	0.032
[HMIM][TF_2_N]	0.031	0.031
[BMIM][TF_2_N]	0.028	0.029
[EMIM][TF_2_N]	0.026	0.028

From a structural perspective, as the alkyl chain length of the imidazolium cation increases from ethyl to decyl, the proportion of non-polar alkyl moieties in the IL increases, leading to enlarged free volume and enhanced hydrophobic microdomains. This structural evolution provides additional accessible solvation space for CO_2_ molecules, thereby improving CO_2_ solubility. Consequently, the experimentally observed enhancement trend is consistent with the key structural descriptors identified by the model, further confirming that the model effectively captures the underlying structure–property relationships governing CO_2_ adsorption in ILs.

In summary, through SHAP-guided IL design and subsequent experimental validation, this work establishes a complete closed-loop encompassing model interpretation, candidate screening and performance verification. The strong agreement between experimental and predicted results demonstrates the robustness and practical applicability of the proposed model, providing a reliable tool for the rational design and rapid screening of high-performance CO_2_-capturing ILs.

### 3.8. Generalization experiment

To further evaluate the effectiveness and generalization ability of the proposed stacking ensemble model, an additional dataset collected by Gao *et al.* [24] was employed to predict the viscosity of ILs. This dataset contains 10 733 data points, involving 852 cations and 240 anions, with temperatures ranging from 253 to 573 K and pressures from 0.6 to 3000 bar. Representative samples of the dataset are listed in table 15.

**Table 15.** Sample data from the IL viscosity dataset.

cation	anion	*T* (K)	*P* (bar)	Lg(viscosity)/mPa.s
[CH_2_^+^]1 = CC = CN(C1)Cc1ccccc1	FC(S( = O)( = O)[N^−^]S( = O)( = O)C(F)(F)F)(F)F	323	1	1.56
CC[n+]1ccn(c1)C	F[B^−^](F)(F)F	304	63.4	1.51
CCCC[N^+^]1(C)CCCC1	FC(S( = O)( = O)[N^−^]S( = O)( = O)C(F)(F)F)(F)F	363	500	1.25
CCCC[N^+^]1 = CC = C(C = C1)C	[B^−^](F)(F)(F)F	323.15	99.8	1.72
CCOC( = O)C[N^+^](CC)(C)C	C(C)OS( = O)( = O)[O^−^]	328	1	2.41
CCn1cc[n+](c1C)C	P( = O)(O)(O)[O^−^]	288	1	2.57

As shown in figure 17, when the stacking ensemble model is applied to this new dataset for IL viscosity prediction, it still achieves the highest prediction accuracy (*R*^2^ = 0.9714, MAE = 0.0431). This indicates that the stacking model effectively captures the nonlinear relationship between Morgan molecular fingerprint descriptors and IL viscosity, demonstrating strong predictive accuracy and robustness. Compared with single models, the hyperparameter-optimized DBO-LGBM and GWO-BPNN models both exhibit improved prediction performance, confirming the applicability and effectiveness of the adopted optimization strategies across different data scenarios. The stable performance of the stacking ensemble in cross-dataset tasks further validates its strong generalization capability.

**Figure 17.**
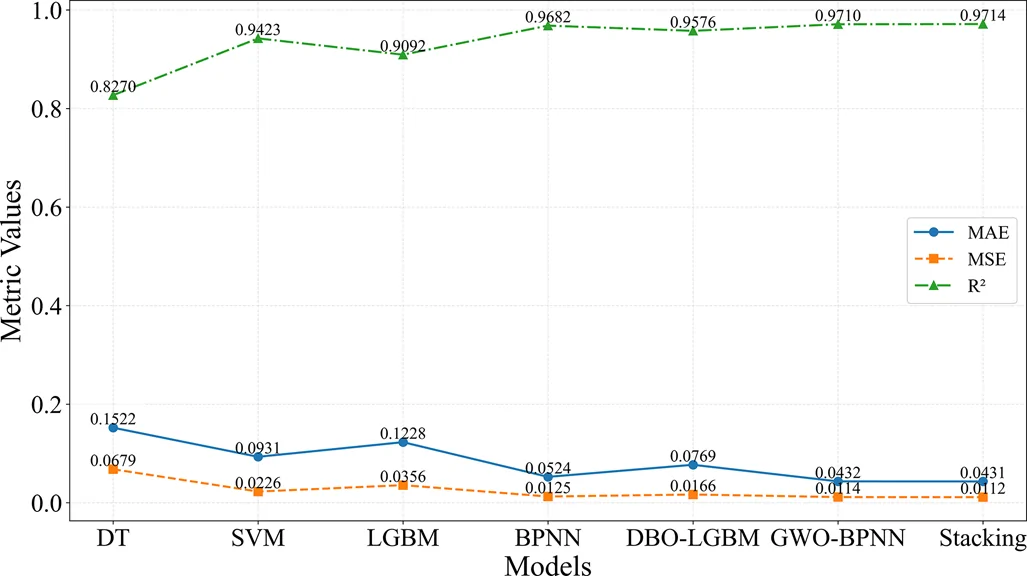
Comparison of IL viscosity prediction performance metrics among different models.

To further evaluate the generalization capability of the proposed model, a more rigorous data-splitting strategy was adopted. Specifically, 10 ILs were selected from the entire dataset as an independent test set, and their cation–anion combinations were ensured to be completely absent from the training set. The SMILES of the ILs in the test set are shown in table 16. This set-up allows for an unbiased evaluation of the model's predictive ability for unseen IL systems. The results show that the stacking model achieves excellent performance on the training set (*R*^2^ = 0.9979, MSE = 0.0008, MAE = 0.0139). More importantly, it maintains high predictive accuracy on the independent test set composed of unseen ILs, with *R*^2^ = 0.9686, MSE = 0.0082 and MAE = 0.0675. Compared with the stand-alone LGBM model (*R*^2^ = 0.9418), the stacking ensemble demonstrates a clear improvement in predictive performance on unseen ILs, indicating that the proposed integration strategy effectively captures the complex nonlinear relationships between IL structure and viscosity and exhibits strong structural generalization capability.

**Table 16.** SMILES of the ionic liquids in the test set.

cation	anion
[CCCCCCn1cc[n+](c1)CCCCCC]	[FC(S( = O)( = O)[N^−^]S( = O)( = O)C(F)(F)F)(F)F]
[CCc1[nH]cc[n+]1Cc1ccccc1]	[FC(S( = O)( = O)[N^−^]S( = O)( = O)C(F)(F)F)(F)F]
[CCCCCC[n+]1ccn(c1)C]	[F[B^−^](F)(F)F]
[CCCCCCN1C = C[N^+^]( = C1)C]	[COS( = O)( = O)[O^−^]]
[CCC[n+]1ccn(c1)C]	[FC(C(C(F)(F)[B^−^](F)(F)F)(F)F)(C(F)(F)F)F]
[CCCCCCCC[n+]1ccn(c1)C]	[[Al^−^](Cl)(Cl)(Cl)Cl]
[CCCC[n+]1ccn(c1)CCCCCCC#N]	[CCCCCCCCCCCCOS( = O)( = O)[O^−^]]
[NCCC[P+](CCCC)(CCCC)CCCC]	[N[C@@H](C( = O)[O^−^])C(C)C]
[CCCC[C@H](C[N+](C[C@@H](CCCC)CC)(C)C)CC]	[[O^−^]C( = O)CC]
[CCCC[PH+](CCCC)CCCC]	[F[B^−^](F)(F)F]

Furthermore, a stratified analysis was conducted to evaluate model performance under different temperature and pressure conditions. As shown in table 17, the temperature-based results indicate that the model maintains high predictive accuracy across the range of 253–574 K. The *R*^2^ values across all temperature ranges exceed 0.92, reaching above 0.98 in the 253–273 and 333–373 K ranges, suggesting that the model accurately captures the temperature dependence of viscosity. Although the high-temperature region (393–574 K) contains only 27 samples, the model still achieves *R*^2^ = 0.9249 and MSE = 0.0121, demonstrating stable performance under high-temperature conditions. The relatively higher error observed in the 293–313 K range (MSE = 0.0210) can be attributed to the large sample size (763) and the greater diversity of IL structures and viscosity distributions in this region. Nevertheless, the *R*^2^ remains at 0.92, confirming the model's robustness.

**Table 17.** Sample sizes and MAE, MSE and *R*^2^ for different temperature intervals on the test set.

temperature (K)	samples	MAE	MSE	*R*^2^
253–293	158	0.0373	0.0051	0.9808
313–333	548	0.0313	0.0094	0.9678
333–353	449	0.0240	0.0031	0.9886
353–373	178	0.0278	0.0019	0.9895
373–393	24	0.0468	0.0059	0.9718
393–574	27	0.0495	0.0121	0.9249

Pressure-dependent analysis further validates the model's applicability over a wide pressure range (0.6–3000 bar). As shown in table 18, except for the low pressure region (0.6–1.1 bar), all other pressure intervals achieve *R*^2^ values above 0.997 with MSE below 0.0005. In the high pressure range (1000–3000 bar), the model still achieves *R*^2^ = 0.9985 and MSE = 0.0003, demonstrating its ability to accurately capture viscosity variations under extreme conditions. The relatively higher error in the low pressure region (0.6–1.1 bar), which contains the majority of samples 1795 samples, can be attributed to the wide structural diversity; however, the model still maintains an *R*^2^ of 0.9675, indicating reliable predictive performance even under complex conditions. Figure 18 shows the bar chart of MSE values across different temperature and pressure intervals.

**Table 18.** Sample sizes and MAE, MSE and *R*^2^ for different pressure intervals on the test set.

pressure (bar)	samples	MAE	MSE	*R*^2^
0.6–1.1	1795	0.0484	0.0134	0.9675
50–100	39	0.0141	0.0003	0.9988
100–1000	198	0.0138	0.0003	0.9987
1000–3000	58	0.0130	0.0003	0.9985

**Figure 18.**
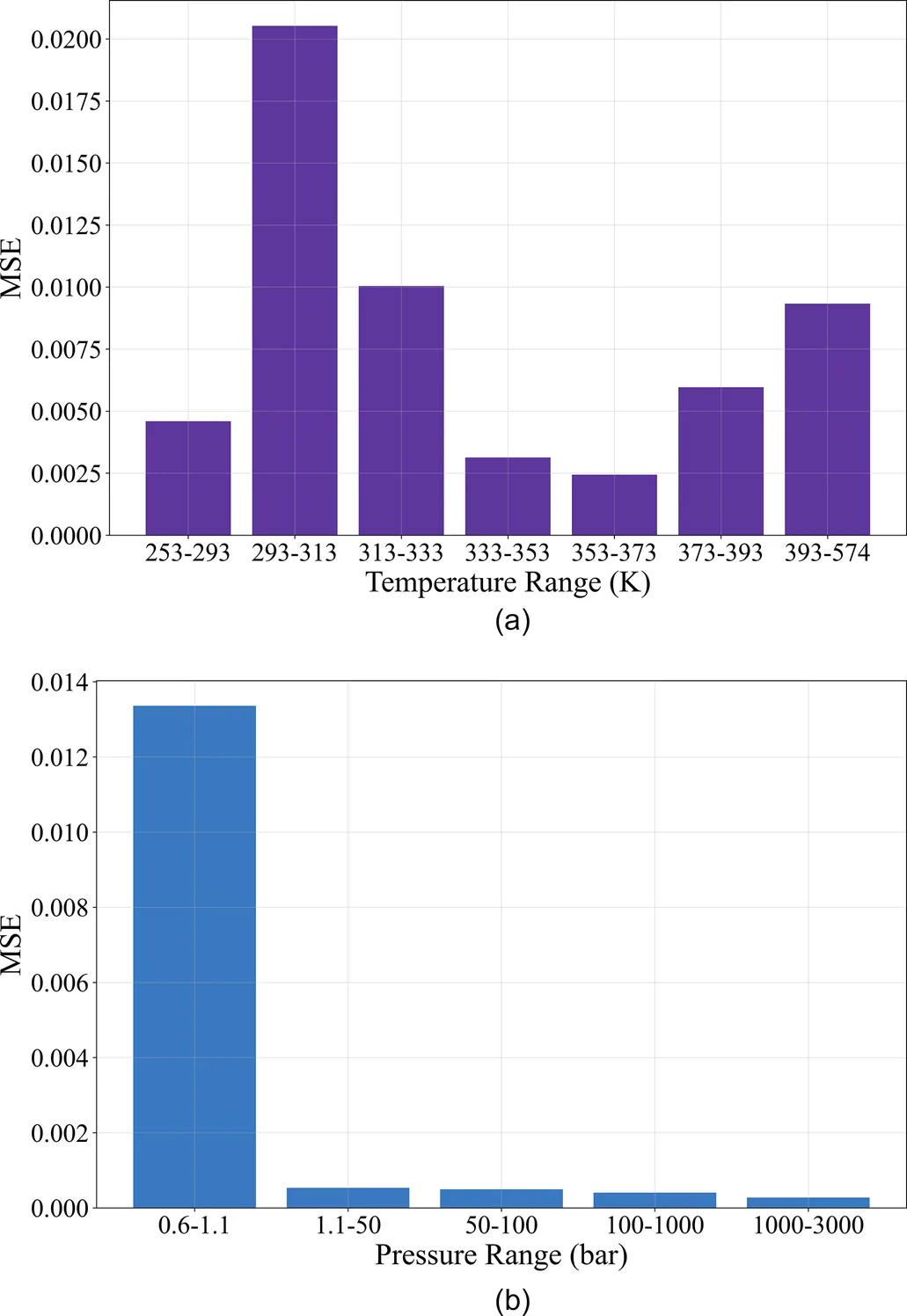
Bar chart of MSE values across different (a) temperature and (b) pressure intervals.

In summary, through unseen IL testing, temperature stratification and pressure stratification analyses, the proposed stacking model is demonstrated to be highly effective in predicting the viscosity of unknown IL systems. Moreover, it maintains stable and accurate performance over a wide operational range of 253–574 K and 0.6–3000 bar. These results collectively confirm that the model possesses strong structural and operational generalization capabilities, meeting the requirements for rapid viscosity prediction and screening of ILs in practical applications.

## 4. Conclusions

In this study, a stacking-based multi-model fusion approach was proposed for predicting the solubility of CO_2_ in ILs. Morgan fingerprints were used as molecular descriptors and combined with experimental parameters, temperature (*T*) and pressure (*P*), as input features, while CO_2_ solubility was taken as the output. DBO-LGBM and GWO-BPNN were integrated as base learners, and LR was employed as the meta-learner to construct the stacking ensemble prediction model. Multiple comparative experiments demonstrated that the stacking ensemble achieved the best overall performance, attaining an *R*^2^ of 0.9888, an MAE of 0.0149 and an MSE of 0.0006 on the test set. Furthermore, SHAP interpretability analysis provided deep insights into the intrinsic mechanisms governing CO_2_ solubility, establishing a quantitative structure–property relationship within the IL system. The influence of the [Tf_2_N] anion was found to be the most significant, followed by the alkyl chain length of imidazolium-based cations. Combining the [Tf_2_N] anion with imidazolium cations bearing longer alkyl chains enables the design of highly efficient CO_2_-absorbing ILs. Extending the proposed stacking framework to the IL viscosity dataset further confirms its strong generalization ability. Overall, this study not only verifies the effectiveness of combining machine learning with Morgan fingerprint descriptors for quantitative property prediction but also provides a transparent, data-driven pathway for understanding structure–property relationships in IL systems. The proposed approach offers a scalable and interpretable method for virtual screening and rational design of ILs for gas-separation applications.

## Supplementary Material



## Data Availability

The data used in this study were obtained from the supporting information of the published article: Gas Solubility in Ionic Liquid (Chemical Reviews, DOI:10.1021/cr300497a), and the complete dataset is provided as supplementary data S1. Data and relevant code for this research work are stored in GitHub (https://github.com/yusiqi-0316/Stacking) and have been archived within the Zenodo repository [36]. Supplementary material is available online [37].
